# Enhancing photovoltaic efficiency in arid climates using cooling strategies

**DOI:** 10.1038/s41598-026-50636-6

**Published:** 2026-05-25

**Authors:** Montaser Abdelsattar, Ola Mostafa A. Saleh, Alaa F. M. Ali, Ashraf N. Eldeen Mourad, Meshari D. Alanazi, Hamdy A. Ziedan, Mohamed Abdelhamid

**Affiliations:** 1 Electrical Engineering Department, Faculty of Engineering, Qena University, Qena, 83523 Egypt; 2 Electrical Engineering Department, Faculty of Engineering, Assiut University, Assiut, 71518 Egypt; 3 Electrical Technology Department, Egyptian German College (EGC), Misr International Technological University (MITU), Assiut, Egypt; 4 Faculty of Industrial and Energy Technology, New Assiut Technological University, Assiut, Egypt; 5 Department of Electrical Engineering, College of Engineering, Jouf University, Sakaka, 72388 Saudi Arabia; 6 Electrical Power Engineering Department, High Valley Institute for Engineering and Technology, Qalyubia, Egypt

**Keywords:** Photovoltaic cooling, Measured power, Active cooling, Spray water, Serpentine cooling, Fame glass, Experimental work, Engineering, Electrical and electronic engineering

## Abstract

This study experimentally investigates the performance enhancement of photovoltaic (PV) panels using different cooling techniques under real outdoor operating conditions. Three cooling approaches are evaluated, including water-spray cooling, serpentine water circulation, and a fame-glass configuration. The objective is to assess their impact on PV surface temperature and electrical performance. Experimental measurements are conducted under similar environmental conditions, and the current–voltage (I–V) characteristics of the PV modules were recorded for each cooling configuration. The results demonstrate that active cooling techniques significantly reduced the operating temperature of the PV module, which consequently improved its electrical performance. Among the tested methods, the water-spray cooling technique achieved the most effective temperature reduction and the highest improvement in power output. In contrast, the fame-glass configuration showed a reduction in electrical power despite lowering the panel temperature, mainly due to optical losses and partial shading caused by the glass frame structure. The findings highlight the importance of selecting appropriate cooling strategies that balance thermal management and optical performance. Overall, the results confirm that effective cooling techniques can enhance PV efficiency and contribute to improved energy production in hot climate regions.

## Introduction

Solar photovoltaic (PV) systems are widely recognized as a key renewable energy technology for sustainable power generation, particularly in regions with high solar irradiance^1,2^. However, the electrical performance of PV modules is strongly influenced by their operating temperature, which becomes a critical limiting factor in hot and arid climates^3,4^. PV module performance is strongly influenced by operating temperature, with 25 °C defined as the reference temperature under standard test conditions (STCs). A decrease in cell temperature below this value generally enhances electrical performance. Although increased solar irradiance leads to higher power output, it may also cause a rise in cell temperature, which can result in a reduction in conversion efficiency. In such environments, a significant portion of the incident solar energy is converted into heat rather than electricity, leading to elevated module temperatures and subsequent efficiency degradation^5^. It has been reported that the efficiency of silicon-based PV modules decreases by approximately 0.4–0.5% for every 1 °C increase in cell temperature above STCs (25 °C)^6,7^. Consequently, in regions such as Upper Egypt, where ambient temperatures can exceed 45–50 °C during summer months, effective thermal management is essential to maintain PV performance, ensure operational reliability, and mitigate long-term thermal degradation^3,8^.

Various approaches have been proposed to address PV temperature rise, including optical concentration, tracking systems, and structural modifications aimed at increasing the incident solar irradiance^7,8^. While these methods can enhance power output, they often intensify thermal stress on PV modules, which may further reduce efficiency and accelerate performance degradation if adequate cooling is not provided^3,4^. Several studies have investigated different PV cooling techniques to enhance electrical efficiency and reduce module temperature. For example, previous works have examined water-spray cooling^9,10^, serpentine water circulation systems^11,12^, and other passive or hybrid cooling approaches^13–16^.

The effectiveness, cost, and suitability of these cooling techniques remain highly dependent on local climatic conditions, particularly in high-temperature regions^3^.

The average efficiency of commercial PV panels is between 15 and 20% under ideal condition, monocrystalline PV can gain efficiency up to 22%, while polycrystalline can gain 18% efficiency^5^.

Under strong solar irradiance conditions, particularly in hot climates, PV modules tend to operate at elevated temperatures, which negatively affect their electrical performance. Therefore, effective cooling is required to limit temperature rise while maintaining high irradiance utilization.

As a result, keeping the units’ temperature below thermal degradation is crucial. It has been shown that, the efficiency of a silicon-based PV panel decreases by 0.5% for each degree Celsius of temperature increase^6^. When the PV panel efficiency reaches its maximum under the given conditions, the surface temperature is around 25 °C^7^. The solar radiation factor states that when more sunlight reaches a PV module’s surface, the module’s efficiency will rise. This is often achieved by using investigation if there are specific research goals or hypotheses, Trackers, lenses, and/or condensers. By lowering the angle of incidence, these methods seek to raise the solar energy density on the PV panel^7,8^. The issue with these techniques is that they cause the temperature of the solar cells to increase over the safe operating range, which can lead to reduced cell efficiency and, in severe cases, even cell damage. As a result, PV modules that are cooled down will perform better, particularly in warm weather^3^.

Efficiency is not over 20% even in the best-case scenario under STCs. Because it depends on several factors, maintaining this efficiency at its best level is the biggest problem. The PV modules must be cooled to maintain the lowest temperature with the maximum radiation.

**Fig. 1:**
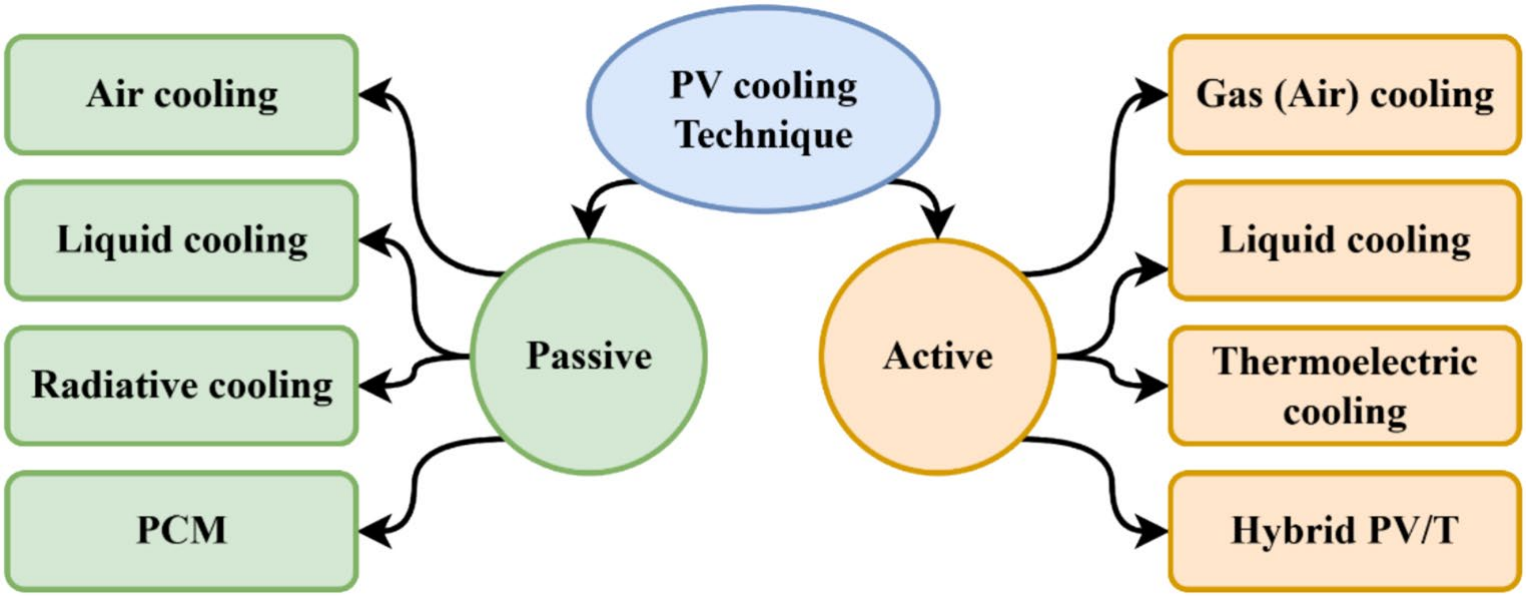
The most popular methods for cooling solar PV panels.

Many researchers have proposed cooling as a means of increasing PV performance and mitigating the impact of cell temperature on performance. A few of the research that have been done to find out more about this subject will be highlighted in this section. Solar panel cooling methods that are most often used are shown in Fig. 1. Cooling systems are categorized into passive and active. Passive cooling solutions don’t require operating energy; however, active cooling solutions require additional energy to cool by extracting heat using pumps, fans, etc. PV modules are rated under standard test conditions, which correspond to a solar irradiance of 1000 W/m^2^ and a cell temperature of 25 °C.

As illustrated in Fig. 1, PV cooling techniques can be broadly categorized into passive and active methods. Passive cooling approaches, including heat sinks and radiative cooling, reduce the operating temperature of PV modules without consuming auxiliary energy by relying on natural heat dissipation mechanisms. Conversely, active cooling techniques, such as spray cooling and hybrid photovoltaic/thermal (PV/T) systems, utilize externally driven fluid flow or coupled thermal–electrical systems to extract heat more effectively, particularly under high irradiance and elevated ambient temperature conditions. Accordingly, Fig. 1 has been updated to reflect a complete and consistent classification of PV cooling techniques discussed in this study.

### Passive cooling techniques

Passive cooling methods are often chosen because they are simple to use and inexpensive. Natural convection serves as the primary cooling mechanism in passive cooling solutions. In addition to natural convection, other uses, including PCMs and thermosiphon systems, could contribute to the cooling^16^.

There are many subcategories of passive cooling techniques are covered in separate discussions. The following are these categories: a) Passive cooling methods based on gas (air). b) Passive cooling methods based on liquid. c) Passive cooling methods based on radiative. d) Passive cooling methods based on phase change material (PCM).

#### Passive Air-cooling techniques

The leading coolant in the passive cooling method, which uses natural convection to decrease the temperature of PV panel, is air. But it does not cool as much as other methods. Gas (air)-cooling methods have so far been the subject of much theoretical and experimental investigation by several researchers.

Theoretically, polycrystalline panels in^17^ and^18^ can be cooled by passive air cooling using devices like heat sinks, which can reduce temperature by 17.61 °C and 16 °C, respectively. For mono crystal panels in^19^, air ducts (flat and curved fins) attached to the back of panel can reduce temperature by 18.09 °C. Additional experimental studies, In Dhahran, Saudi Arabia, aluminium fins with a nano coating placed on the back of polycrystalline panels lowered temperature by 8 °C and increased efficiency by 2.88^20^. In Tiruchirappalli, India, fin-attached heat sinks lowered temperature by 9.45 °C and increased efficiency by 1.072^21^. In Selangor, Malaysia, polycrystalline panels cooled by both longitudinal and lapped fin heat sinks, which decreased temperature by 22.7 and 14.28 °C, respectively, and increased efficiency by 12.33 and 8.86, respectively^22^.

#### Passive liquid cooling techniques

Water and other fluids, including chemical fluids like glycol, as well as nanofluids, are the primary coolants employed in these passive cooling methods to reduce the PV panel’s operating temperature. These methods use gravity, thermosiphon effects, heat pipes, floating, and other natural fluid circulation to cool PV panels without requiring any additional electricity. Compared to gas (air)-based passive cooling approaches, these methods have a superior cooling effect. In theoretical study^23^, polycrystalline panels are cooling using both Heat sink based on passive air, and Thermosiphon loop based on passive water, the temperature decreased by 25 °C, 36.5 °C, and the efficiency increased by 30%, and 41.5%, respectively.

Theoretically, a solar-driven rainwater cooling system was studied for cooling poly crystal panels; the temperature decreased by 19 °C and the efficiency increased by 8.3%^24^, in other study the porous channel cooling method increased the efficiency by 4.17%^25^.

Experimentally in Karnataka (India) applied Evaporative cooling, and Gravity assisted flow cooling techniques for cooling poly crystal, The results showed decreases in the temperature by 15.9 °C, and 8.7 °C and increases in efficiency by 45%, and 33%, respectively^26^. After using floating cooling with fin assistance, the efficiency of polycrystalline panels at Port Said, Egypt, increase by 22.24%^27^. In Baghdad, Iraq, monocrystalline solar panels’ efficiency increased by 9.7% following the use of evaporative cooling, which dropped the temperature by 18.3 °C^28^. At Thuwal (Saudi Arabia), following the application of a solar-driven PV cooling technology that lowered the temperature by 8 °C, their efficiency increased by 47%^29^.

#### Passive phase change material (PCM) cooling techniques

Through the phase transition from solid to liquid in temperature latent, PCMs store thermal energy. By switching phases, PV panels may be cooled without using any energy. More cooling occurs than with passive air-based cooling. Theoretically, Authors have used PCMs as coolant materials, in^30^ using RT27 increased the efficiency By 12%, in^31^ using RT42 paraffin wax decreased temperature by 32 °C, and in^32^ using RT35HC paraffin wax decreased temperature by 24.9 °C and increased efficiency by 11.03%.

On the other side, many research works have applied experimentally PCM as a cooling technique, OM47 was employed as PCM cooling in Karnataka, India, to cool polycrystalline PV panels, this method reduced temperature by 8.1 °C and raised efficiency by 13%^26^. In Jahrom, Iran, the poly-crystalline panels were cooled with PEG1500 passive PCM cooling in addition to a heat sink. This resulted in a 28 °C drop in temperature and a 3% gain in efficiency^33^. Paraffin is used for cooling poly-crystalline panels in Dezful (Iran) that leads to decrease temperature by 6.8 °C and increase efficiency by 12.5%^34^. When using Glauber salt to cool monocrystalline panels in Tamil Nadu, India, the temperature decreased by 31 °C and the efficiency increased by 11.5%^35^.

### Active cooling techniques

Energy is used throughout the cooling process in active cooling systems. Forced convection is the primary cooling mechanism in active cooling systems. Three distinct categories are used to categorize active cooling methods. These consist of a) Active Gas (air) cooling techniques b) Active Liquid cooling techniques c) Active Thermoelectric cooling techniques.

#### Active gas (air) cooling techniques

In active cooling approaches, air is employed as the primary coolant to drive convection and lower the temperature of the PV panel. But compared to gas (air) cooling methods, there is more cooling.

Studies like^36^, which used both active fan cooling and passive nature cooling in an experimental to cool mono-crystalline panels, compare passive and active air cooling. The results showed a 33.33% reduction in the panel’s performance assessment cost.

A 50 kW poly-crystalline PV experimental system in Mathura, India was cooled utilizing both passive natural cooling and active artificial cooling which led to an increase in the performance of the cell to 95.9% and 98.2%^37^.

The forced air cooling was applied on the front side of the polycrystalline panels in Ismailia, Egypt, the temperature decreased by 7 °C and the efficiency increased by 2.29%^38^. Whereas active air cooling with a channel under the monocrystalline panels is used experimentally in Tehran, Iran, the temperature drops by 5 °C and the efficiency increases by 2.6%^39^.

In Benha, Egypt, researchers conducted an experimental comparison of fans and blowers for forced convection cooling on the rear side of monocrystalline PV panels. The results showed that efficiency increased by 3.9% and 7%, respectively, and temperatures decreased by 5.4 °C and 9 °C, respectively^40^.

#### Active liquid cooling techniques

Water and other fluids, including chemical and nanofluids, are the most common coolants utilized in this active cooling method to lower panel temperatures. This approach cools the panels by forcing the fluids to circulate using energy. This method provides a greater quantity of cooling than passive liquid cooling methods.

Techniques for active liquid cooling are researched. Theoretically^41^, addressed the effects of jet-impingement the cooling with SiC-water nanofluid on panels, resulting in a temperature reduction of 32.1 °C. Similarly^42^, examined the effects of water spraying at the back side of the panels as a cooling method, resulting in a temperature reduction of 10◦C and an increase in efficiency of 7.3%.

However, they are used experimentally in many studies, including the following: at Alcala de Henares (Spain), where the temperature lowered by 14 °C and efficiency improved by 9.8% after using heat exchanger at the back side of the poly-crystalline panels^43^; in^44^ at Ankara (Turkey) where the temperature lowered by 1.88 °C and efficiency increased by 13.69%, using water film that flowing on the upper side of the panel; and in^45^ using spraying of water on the upper side of the monocrystalline panel that lead to increase efficiency by 15.73%.

Nanofluid was used to improve the cooling systems as follows: in^46^ at Xi’an (China), nanofluid including 2% Al_2_O_3_ combined with heat pipe groundwater and spiral pipe was used to improve the cooling systems for mono-crystalline panels; and in^47^ at Baghdad (Iraq), nanofluid with Zn-H_2_O combined with a heat exchanger on the back of the panel was used to cool mono-crystalline panels, resulting in a decrease in temperature of 18 °C and an increase in efficiency of 7.8%.

#### Active thermoelectric cooling techniques

This active cooling technique lowers the temperature of PV panels via the thermoelectric effect. Electric energy is used to cool using active thermoelectric cooling technology. With this method, electrical current is used to cool the panels. The junction of two different wires cooled when a little current was sent through it. The Peltier effect is a phenomenon that is fundamental to thermoelectric refrigeration. Heat is taken in from the medium to be cooled and rejected in a warmer environment in this refrigeration circuit. The net electrical input that is still available is the difference between these heat levels. Compared to other active cooling strategies, this method provides less cooling^48^.

A thermoelectric module installed on the back of a photovoltaic panel is used to theoretically analyse the cooling effects. The cooling improved efficiency by 18%^48^. However, an experimental study on how a thermoelectric module mounted to the back of the panel for cooling the panel. The corresponding cooling strategy reduced the temperature by 35 °C and increased efficiency by 18%^49^.

This research work tested experimentally in the Renewable Energy Laboratory at Assiut University, Egypt. The experiment was done under high temperatures in the summer months which reached 50 °C. So that three different cooling systems are chosen to deal with high temperature and selected the most effective. These techniques are selected between passive and active categories. They are spray water, serpentine with water, and fame glass.

The main goal of the paper is to search for effective cooling systems that deal with PV panels in hot places.

Search for the benefits and drawbacks of using spray water for cooling PV systems. Investigate the advantages and disadvantages of employing a serpentine water-cooling system for PV panels. Research the properties and applications of “fame glass” in the context of PV cooling, considering if it might be a specific type of glass or “fame glass”.

The fame-glass technique represents a passive cooling approach in which a conventional glass sheet is coated with a thin semi-shading layer that allows partial transmission of solar radiation. Unlike reflective coatings or advanced spectrally selective films reported in previous studies, the fame-glass used in this work relies on commercially available treated glass similar to automotive tinted glass. This approach provides partial irradiance reduction and thermal mitigation without complex fabrication processes, offering a simple and practical passive cooling alternative for photovoltaic applications.

Previous studies have reported significant performance improvements using active water-based cooling techniques for PV modules. Spray cooling methods have demonstrated efficiency enhancements ranging from approximately 7% to 24%, with corresponding surface temperature reductions of 10–22 °C under high irradiance conditions^42,45^. Similarly, serpentine or channel-based water cooling systems have achieved temperature reductions of 15–25 °C and efficiency improvements between 9 and 25%, depending on flow configuration and operating conditions^43–45^.

In contrast, passive cooling approaches based on glass covers or shading elements have been shown to reduce PV surface temperature by 8–12 °C; however, several studies report substantial power losses, in some cases exceeding 30–60%, due to irradiance attenuation caused by reflection, refraction, and partial shading effects^24,26^. These findings indicate that while passive glass-based cooling can lower operating temperature, it often introduces a significant trade-off between thermal mitigation and electrical output.

Accordingly, the present study experimentally compares spray cooling, serpentine water cooling, and glass-frame cooling under identical outdoor conditions to quantitatively assess their thermal and electrical performance trade-offs in hot arid climates.

The motivation of this work is to analyze the factors influencing the effectiveness of different PV cooling techniques and to evaluate their suitability for hot climatic regions:

1. Analyse the characteristics of the climate in Asyut Governate, Egypt, and how these three cooling techniques might be particularly suitable for this environment.
2. Compare and contrast the expected cooling performance, cost-effectiveness, and complexity of implementation for each of the three techniques: spray water, serpentine water cooling, and “fame glass”.
3. Look for studies that compare active cooling methods like spray water and serpentine water cooling with passive methods potentially represented by “fame glass” to understand the reasons for including both categories.
4. Investigate if there are specific research goals or hypotheses in the mentioned research that necessitated the selection of these particular three cooling methods.
5. Explore research publications or studies from the Renewable Energy Laboratory at Assiut University in Egypt that discuss the rationale behind selecting spray water, serpentine water cooling, and “fame glass” for their PV cooling experiments.

The rest of the paper is organized as follows: Section "Methodology" presents the proposed methods for cooling PV panels, then Section "Measurements" describes tools which used in measurement, and the steps of measuring power, the results and their discussion displayed in Section "Results", and finally, the conclusion of the proposed method in Section "Limitations of the study".

Asyut Governate, located in Upper Egypt, is characterized by an arid climate with prolonged periods of high solar irradiance exceeding 1000 W/m^2^ and ambient temperatures that frequently surpass 45–50 °C during summer months. Under such conditions, PV modules experience severe thermal stress, leading to pronounced efficiency degradation and accelerated aging. Therefore, cooling techniques selected for this study were required to be effective under extreme temperatures, technically simple, and adaptable to real outdoor installations.

Spray water cooling was selected due to its high heat removal capability through combined convective and evaporative mechanisms, which makes it particularly effective during peak temperature and irradiance periods. This method is well suited to regions where water is locally available and rapid temperature reduction is required. In contrast, the serpentine water-cooling technique was chosen to represent a more controlled and water-efficient active cooling approach, capable of providing stable thermal regulation and improved average power output throughout the operating day.

The glass-frame configuration was included as a passive cooling technique to evaluate a low-cost, energy-free thermal mitigation strategy that relies on partial shading and reduced radiative heat gain. Although such passive approaches may reduce module temperature, their suitability in high-irradiance arid climates remains uncertain due to potential optical losses. Accordingly, the inclusion of the glass-frame system enables a direct experimental assessment of the trade-off between temperature reduction and irradiance attenuation under the specific climatic conditions of Assiut.

Despite the extensive body of research on photovoltaic cooling techniques, a clear gap remains in experimentally comparing active and passive cooling strategies under identical real outdoor conditions in extremely hot arid climates. Many previous studies focus on individual cooling methods or rely on controlled laboratory environments, which limits the direct comparability of their results and their applicability to real operating conditions.

Motivated by the harsh climatic conditions of Upper Egypt, this study aims to provide a side-by-side experimental evaluation of three representative cooling techniques—spray water cooling, serpentine water cooling, and glass-frame cooling—using identical photovoltaic modules operating simultaneously under the same environmental conditions. The novelty of this work lies in its systematic assessment of the thermal–electrical trade-offs between highly effective active cooling methods and low-cost passive cooling approaches, with particular emphasis on water consumption, temperature reduction, and electrical performance degradation.

The findings of this study offer practical insights for selecting suitable PV cooling strategies in hot arid regions, supporting the deployment of more reliable and efficient photovoltaic systems under extreme environmental conditions.

## Methodology

This section of the study introduces three techniques for cooling photovoltaic panels. PV panels have been established on the laboratory rooftop, facing south at a tilt angle of 30 degrees, in line with the site’s latitude. Before the readings, every PV panel was maintained clean. The datasheet data is listed in .

Table 1.

**Table 1:** Technical data of PV module at standard test conditions; AM = 1.5, G = 1000 W/m^2^, cell temperature T = 25 °C.

Item	Unit	Value
Peak power	*P*_*max;*_ Wp	175 $\pm$ 10%
Max. power current	*I*_*mp*_; A	5.0
Max. power-voltage	*V*_*mp*_; V	35.0
Short circuit current	*I*_*sc*_; A	5.4
Open circuit voltage	*V*_*oc*_; V	44.4
Dimension	Mm	808 × 1580 × 35
Temperature coefficient for	*Pmax*; % /°C	-0.50
Temperature coefficient for	*V*_*oc*_; % /°C	-0.35
Temperature coefficient for	*I*_*sc*_; % /°C	0.09
Number of cells in series	–	72
Number of cells in parallel	–	1

**Fig. 2:**
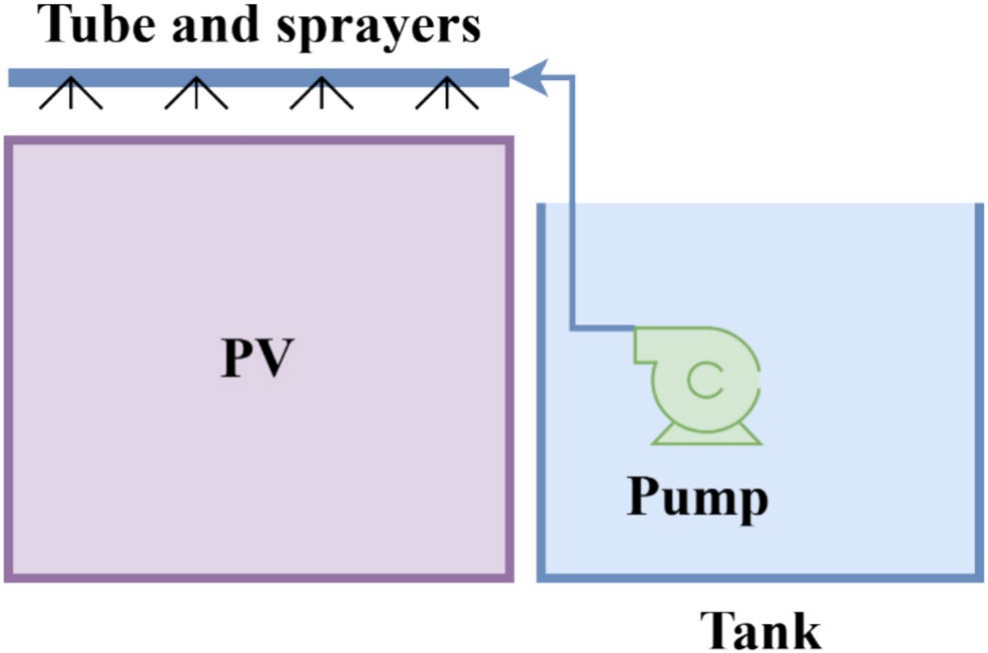
The schematic form of the spray water technique*.*

**Fig. 3:**
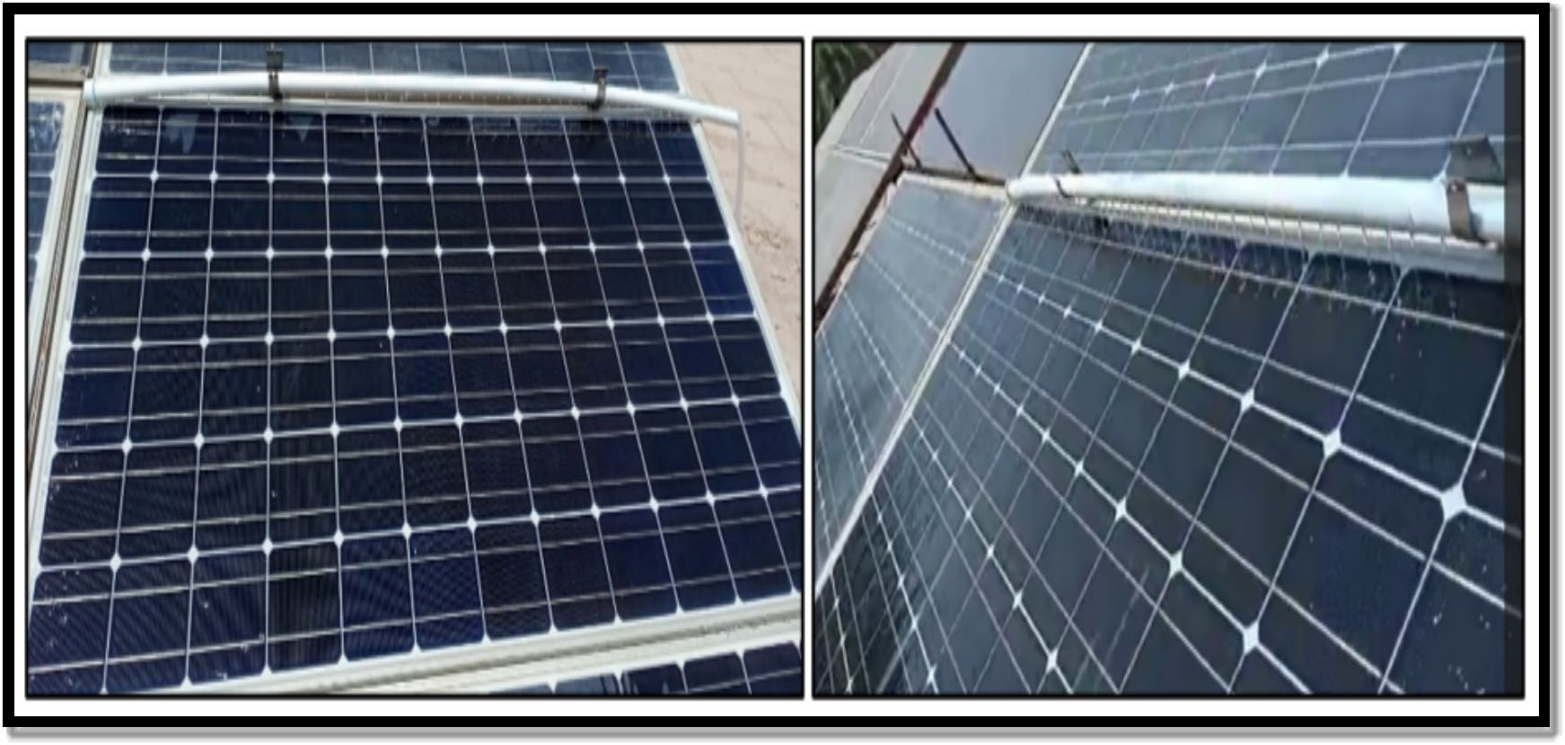
The water-spray system for cooling PV panels.

The first technique, known as the Water-spray technique. The spray cooling system was designed as an open cycle system and had a pump with 45 watts, and maximum flow rate 1000 Liter / Hour, but the real flow is 2 Liter / minute, and provided with a 50-L storage tank as shown in Fig. 2. Dimensions of spray system consist of a 1-cm diameter of PVC tube which is fixed in the top of PV module with holes separated 2.5-cm apart. This PVC tube connected with a tank of water with a pump and control unit. The spray system works to spray water over the panel for 5 min at a time, pausing for 10 min and repeating all day or time of work, from 9:00 am to 3:00 pm, as shown in Fig. 3.

For the spray cooling technique, water was supplied at a flow rate of approximately 2 L/min, and the system operated intermittently for 5 min followed by a 10-min pause. Accordingly, each spraying session consumed approximately 10 L of water. This operating strategy was selected to balance effective evaporative cooling with water consumption efficiency, particularly under arid climatic conditions.

**Fig. 4:**
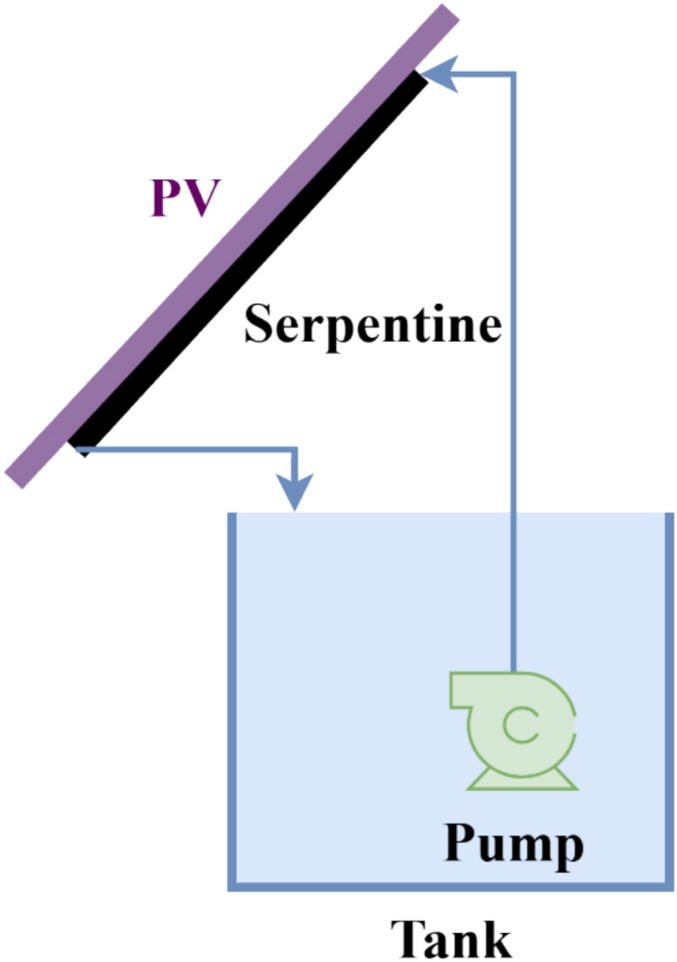
The schematic form of the serpentine cooling technique.

The spray system consisted of a PVC pipe with a diameter of 1 cm, fixed along the upper edge of the PV module frame. The pipe was perforated with evenly spaced holes at 2.5 cm intervals, allowing water to be uniformly distributed across the module surface. This configuration ensured homogeneous spray coverage and effective heat removal over the entire panel area, Fig. 4.

To mitigate any potential shading effects, the PVC pipe was positioned outside the active photovoltaic cell area and aligned with the module frame. Owing to its small diameter and peripheral placement, the shading impact on the incident solar irradiance was negligible and did not measurably affect the electrical output of the panel during operation.

The second cooling approach, referred to as the serpentine water-cooling technique, employs a closed-loop liquid circulation system installed on the backside of the photovoltaic module. Water was circulated through the serpentine at a constant flow rate of approximately 2 L/min. This flow rate was selected as a compromise between achieving sufficient convective heat removal and minimizing pumping power consumption, based on preliminary trials and the pump operating characteristics.

The cooling system was driven by a 45 W pump with a maximum rated capacity of 1000 L/h and connected to a 50-L storage tank. To enhance the cooling potential during high-temperature conditions, the water in the tank was pre-cooled using ice, maintaining the inlet water temperature below 10 °C. Water circulated continuously through the serpentine from 9:00 am to 3:00 pm, corresponding to the full operational period of the PV modules.

**Fig. 5:**
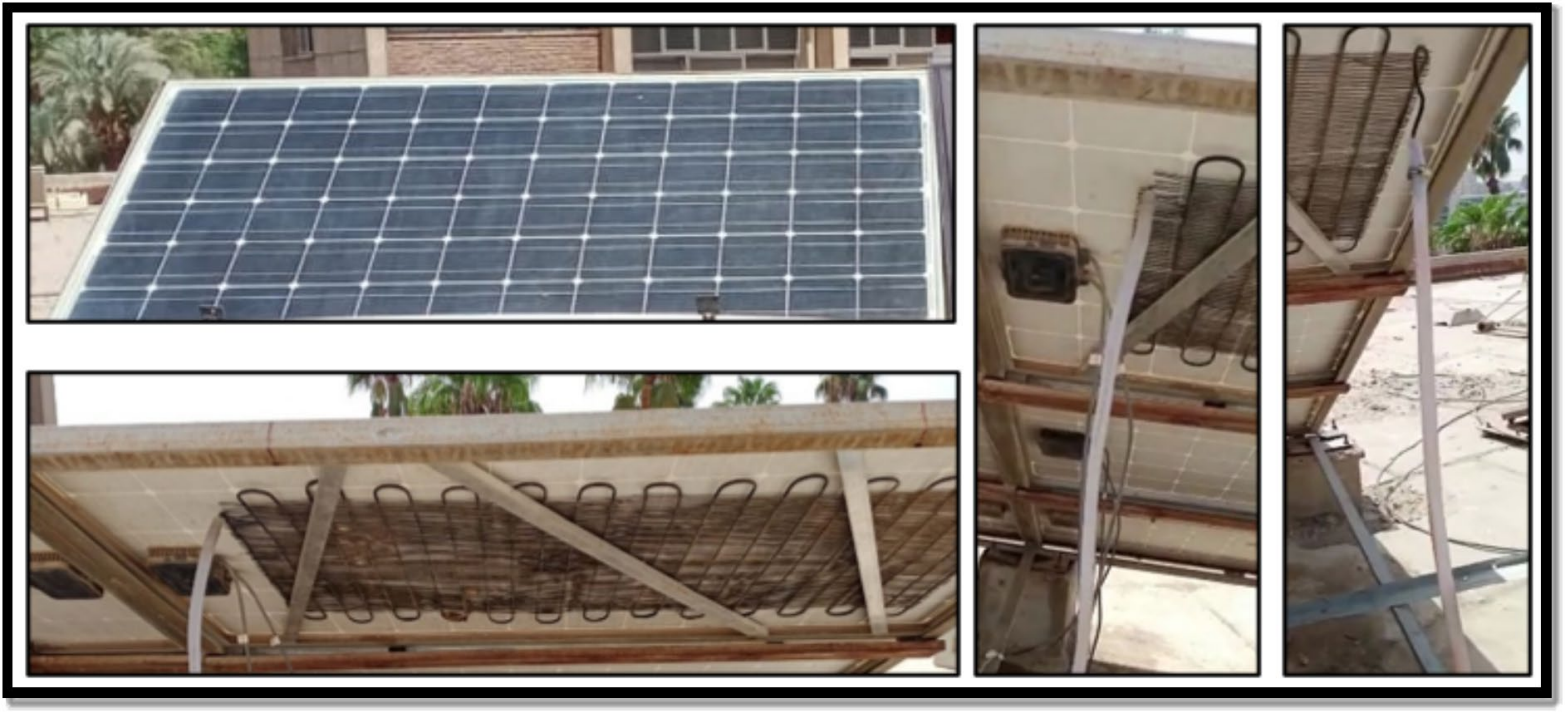
Iron-serpentine with water technique for cooling PV panels.

The serpentine heat exchanger was fabricated from a copper tube with a diameter of 0.5 cm and a total length of approximately 5 m, covering nearly 80–85% of the PV module backside area. The serpentine was firmly fixed directly onto the rear surface of the PV panel using mechanical clamps to ensure intimate thermal contact. This direct attachment minimized thermal contact resistance and enabled efficient heat transfer from the PV module to the circulating water, Fig. 5.

**Fig. 6:**
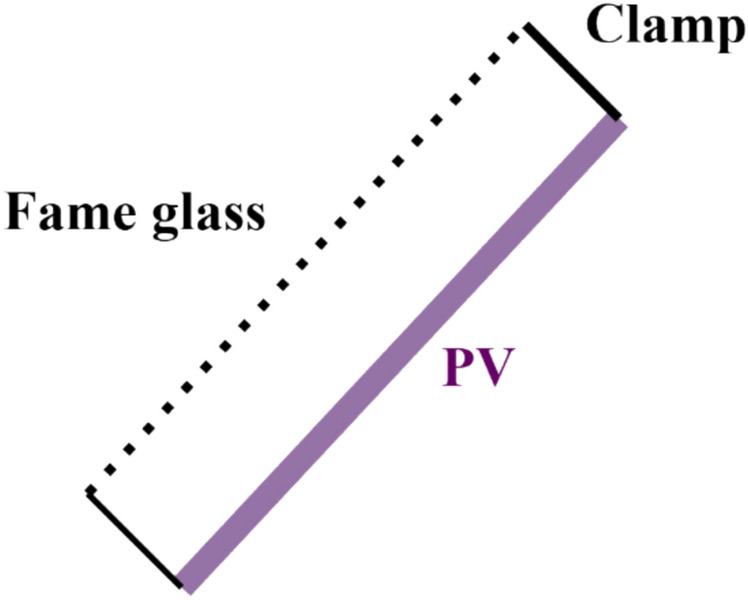
The schematic form of the fame glass technique.

The fame-glass was implemented by placing a transparent glass sheet above the photovoltaic module with a uniform air gap of approximately 2 cm. This separation distance was selected to allow natural air circulation between the glass and the PV surface while reducing direct solar heat gain through partial shading and radiative attenuation, Fig. 6. The term “fame-glass” refers to a conventional glass sheet coated with a thin semi-shading layer that partially transmits solar radiation. This type of treated glass is similar to automotive tinted glass, which is commonly used to reduce solar heat gain while allowing partial sunlight transmission. In this study, the fame-glass layer is used as a passive cooling approach to limit excessive irradiance and thermal loading on the photovoltaic module.

The glass used in this configuration had an average solar transmittance of approximately 85–90% in the visible spectrum. Despite its relatively high transmittance, the presence of the glass inevitably reduced the effective irradiance reaching the PV surface due to reflection and refraction losses.

**Fig. 7:**
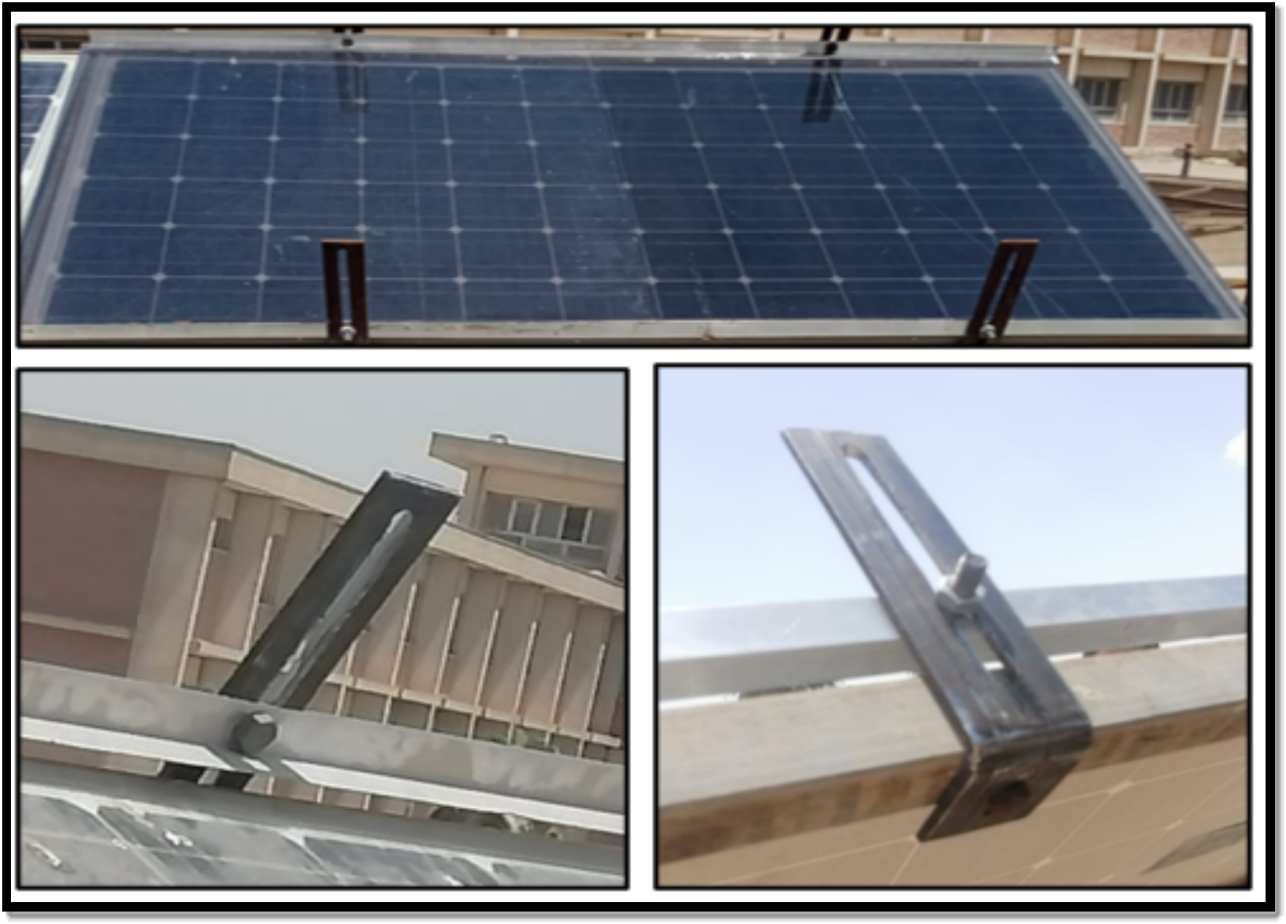
Fame-glass technique for cooling PV panels.

The glass frame was mechanically supported using longitudinal mounting clamps, as shown in Fig. 4. These clamps introduced localized shading on the PV surface, which contributed to additional power losses beyond those associated with optical attenuation alone. While this shading effect affects direct comparability with the actively cooled configurations, it realistically represents practical installation constraints often encountered in low-cost passive shading systems. Consequently, the glass-frame technique was intentionally included to experimentally assess the thermal–electrical trade-off and the limitations of passive shading-based cooling approaches under real outdoor conditions, Fig. 7.

## Measurements

In this section, tools these used in measurements are presented, then steps for measuring output power are described in specific subsection.

### Measurements tool

**Fig. 8:**
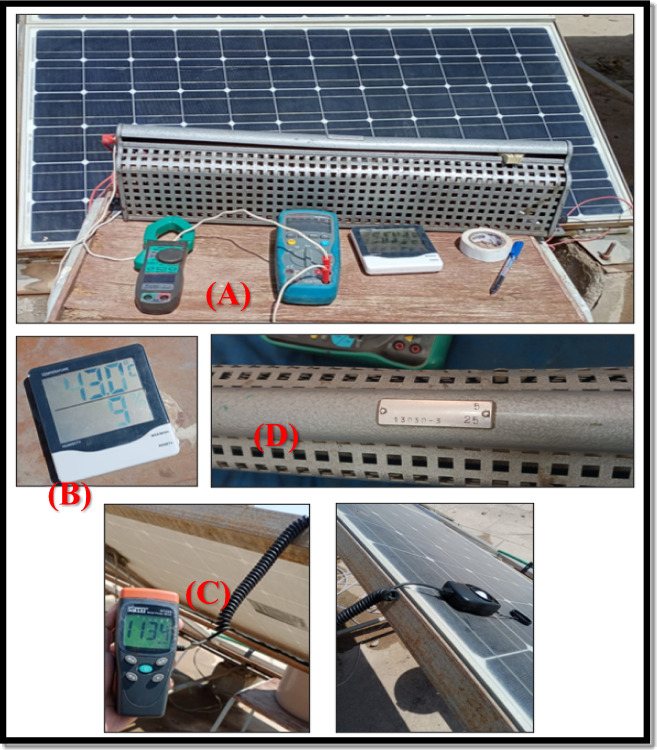
Measurement instruments for PV panel voltage, current, temperature, and radiation; Renewable Energy Laboratory at Assiut University, Egypt.

Panel voltage (V), current (I), temperature (T^o^ C), and solar irradiance (w/m^2) were measured every hour from 9:00 am to 3:00 pm for six weeks. The measurement instruments are displayed in Fig. 8: Fig. 8**-A** shows both the voltmeter and clamp meter for reading voltage and current, respectively; Fig. 8**-B** shows the digital thermometer for temperature measurement; Fig. 8-C shows the light meter (lux-meter) for solar radiation measurement and; Fig. 8**-D** shows the variable resistance.

The current–voltage (I–V) characteristics of the photovoltaic modules were obtained using a variable resistive load. During each measurement cycle, the resistance value was gradually increased from near-zero (short-circuit condition) to a maximum value of 25 Ω (open-circuit condition). The resistance adjustment was performed in 19 discrete steps, ensuring sufficient resolution to accurately capture the full I–V curve.

At each resistance step, the corresponding current and voltage values were recorded after allowing a short stabilization period to ensure steady-state operation. This stepwise loading method enabled consistent I–V curve acquisition at each hourly measurement interval and ensured comparability between different cooling configurations under identical environmental conditions.

Table 2 listed the accuracy of the measurement instruments used in the experimental work.

**Table 2:** Accuracy of the measurement instruments used in the experimental work.

Devices	Accuracy	Resolution	Range
Voltmeter	± 0.6% rdg ± 4dgt ± 0.6% rdg ± 4dgt	**-**	400 mV : 400 V 600 V
Clamp ammeter	± 2.0% rdg ± 4dngt ± 1.0% rdg ± 4dgt ± 1.6% rdg ± 4dgt	**-**	400 : 4000 μA 40 : 400 mA 4 : 10 A
Digital TFA Thermo-Hygrometer	± 1.0 °C & °F and ± 2% midrange ± 4% elsewhere	0.1 Temperature and 1.0% relative humidity	-14 to + 140°F, -10 to + 60 °C
HT 204 Solar Power meter	> between ± 10 W/m^2^ and ± 5% rdg and ± 0.38 W/ m^2^/°C from 25 °C	1.0 W/m^2^	1–1999 W/m^2^

### Measurements and readings steps

First measurements are recorded of both the ambient temperature and sun irradiation readings, using digital thermometers and lux meters were linked.

Second, about panel:

i. For determining the short circuit current, immediately connect the panel’s two outer ends to form a short circuit, and then use the clamp for recording the current’s value.
ii. The voltmeter is connected to both terminals of the cell, for reading the open circuit voltage and recording its value.
iii. The clamp meter is wrapped around the wire to determine the current value, and the voltmeter is connected in parallel with the PV cell’s terminal to measure the output voltage value. The variable resistance is set to a low value and connected to the two outer ends of the PV panel.
iv. Gradually raising the resistance value to create the I-V curve for PV panel.

Third, proceed these steps with all Panels by repeating the earlier processes.

Fourth, compared the cooling approaches using the measurement reading.

Finally, the ratio of the measured maximum output power (produced) of PV panels with its input power (amount of solar irradiance striking the array) is used to determine the panel’s efficiency^50^. The power gain % (${\eta}$) obtained due to the application of the cooling technique relative to the reference PV panel (${P}_{max,Panel}$) is calculated as follows:

1 $${\eta}=\frac{{P}_{max,Panel }- {P}_{max,Ref}}{{P}_{max,Ref}} * 100\mathrm{\%}$$

where, ${P}_{max,Ref}$ is the maximum output power measured with Reference Panel A.

This modification clarifies that the parameter represents the relative improvement in power output rather than the intrinsic PV efficiency.

### Uncertainty analysis and measurement reliability

To assess the reliability and repeatability of the experimental measurements, an uncertainty analysis based on standard deviation and error propagation was conducted. The standard deviation (σ) of a measured quantity was defined as follows:

2 $$\sigma =\sqrt{\frac{1}{\left(1-N\right)}\sum_{i=1}^{N}{\left(\overline{x }-{x}_{i}\right)}^{2}}$$

where, ${x}_{i}$​ represents the individual measured values, $\overline{x }$ is the mean value, and N is the number of measurements.

However, due to the outdoor nature of the experiment and the continuously varying environmental conditions (solar irradiance, wind speed, and ambient temperature), repeated measurements under identical conditions were not always feasible. Therefore, the uncertainty of the electrical power output was quantified using the propagation of uncertainty method, based on the measured voltage and current and their respective instrument accuracies.

The combined uncertainty in power output (ΔP) was calculated using:

3 $$\Delta P=\sqrt{{\left(\Delta V\frac{\partial P}{\partial V}\right)}^{2}+{\left(\Delta I\frac{\partial P}{\partial I}\right)}^{2}}$$

where, P = VI and ΔV and ΔI represent the uncertainties in voltage and current measurements, respectively. This approach provides a reliable quantitative estimate of measurement uncertainty and ensures the comparability and credibility of the reported experimental results.

### Justification of measurement interval and data reliability

The outdoor experimental measurements were conducted using a manual I–V curve acquisition approach, which requires sufficient time to ensure stable operating conditions and accurate electrical characterization of each PV panel. Consequently, an hourly measurement interval was adopted to allow system stabilization and complete I–V data acquisition for all tested configurations under identical environmental conditions.

Unlike short-interval point measurements, each recorded data point in this study represents a full electrical characterization cycle rather than an instantaneous snapshot. Transient environmental fluctuations, such as short-term wind gusts or brief cloud passages, were inherently averaged during the measurement process. Furthermore, data reliability and consistency were ensured through uncertainty propagation analysis based on the specified accuracies of the measurement instruments, allowing anomalous deviations to be quantitatively bounded.

Accordingly, the selected measurement interval provides a reliable representation of the thermal and electrical performance trends of the PV panels under real outdoor conditions, while maintaining experimental feasibility and repeatability.

The electrical performance of the PV module was monitored using a calibrated digital multimeter to measure voltage and current, while the maximum output power was determined from the measured I–V characteristics. In addition, a temperature sensor was used to record the PV surface temperature, and a solar power meter was used to measure the incident solar irradiance during the experimental tests. All measurements were conducted under similar environmental conditions to ensure consistency and comparability between the different cooling configurations.

## Results

The study was done at Assiut University in Egypt in the Renewable Energy Laboratory. To guarantee maximum energy output year-round. The six-week period from 1^st^ September to mid-October 2022 saw the recording of results, which were checked hourly between 9:00 am and 3:00 pm. practically, to research the best PV cell cooling methods.

The thermal impact of several PV panel cooling methods is displayed. A few comparisons are shown, including the experimental I-V curves of each cooling method used during the day, the experimental output power of solar PV panels using various cooling techniques during the day, and a comparison of the output power of solar PV panels at various temperatures during the day using various cooling techniques.

**Fig. 9:**
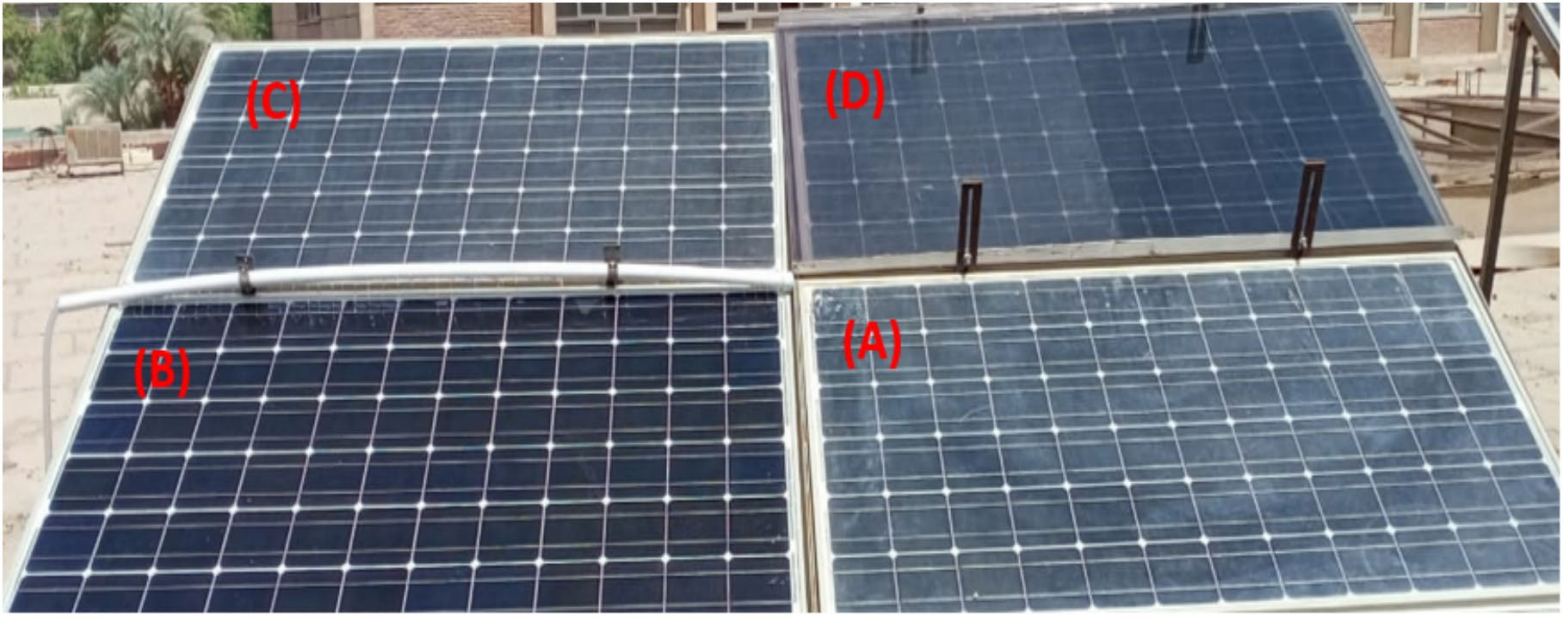
PV panels with different cooling techniques, (**A**) Reference panel; (**B**) Water-spray technique; (**C**) Iron-serpentine with water technique; and (**D**) Fame-glass technique.

In this study, four identical photovoltaic panels are used, each with a different cooling technique. As seen in Fig. 9, as follows:

- Panel A: Reference Panel, Fig. 9-A, which was used to examine the impact of cooling methods on other PV panels but did not have any cooling techniques.
- Panel B: As seen in Fig. 9-B, this PV panel cooling method uses water spraying.
- Panel C: Water-based iron-serpentine process, as illustrated in Fig. 9-C.
- Panel D: The fame-glass method, presented in Fig. 9-D, employs a different fame-glass.

### Experimental results and discussions

The experimental results were measured over 3 days on 1^st^ September, 15^th^ September, and 1^st^ October. Results of each day have a table that includes columns as follows (Time) has an hour of readings, (Temperature) has the reading of ambient temperature in Celsius degrees. (Irradiance) has the irradiance of solar in watts per meter square, and the maximum power per each panel in watts as (Panel (A)) (Panel (B)) (Panel (C)) (Panel (D)), respectively. This table also has the maximum efficiency (Max η), the maximum value (Max) and average (Mean) values of each column.

The following figures are displayed the I-V and P–V curves for the four panels included in the study. The black curves correspond to the reference panel (Panel A), which did not have any cooling techniques; the red curves correspond to the panel that applied the spray water technique (Panel B); the blue curves correspond to the panel that applied the iron-serpentine with water technique (Panel C); and the purple curves correspond to the panel that applied the fame-glass technique (Panel D).

#### PV panel temperature variation under different cooling techniques

The operating temperature of PV panels is a key factor influencing their electrical performance and overall efficiency, particularly under hot climatic conditions. In this study, the surface temperature of each PV panel was measured hourly at each data collection time step (from 9:00 am to 3:00 pm) using a calibrated digital thermometer, simultaneously with the electrical measurements.

Table 3 illustrates the temporal variation of PV panel temperature for the reference panel (Panel A) and the panels equipped with different cooling techniques (Panels B, C, and D). As expected, the reference panel exhibited the highest operating temperature throughout the day, reaching peak values during midday hours due to high solar irradiance and elevated ambient temperatures, which in some cases exceeded 50 °C.

**Table 3:** A reading of each panel during day work (September 1^st^, 2022).

Time	Temperature, °C	Irradiance, w/m^2^	Output power, W			
			Panel (A)	Panel (B)	Panel (C)	Panel (D)
09 am	46	870	97.15 ± 1.66	102.81 ± 1.76 (5.83%↑)	114.17 ± 1.95 (17.52%↑)	44.57 ± 0.76 (54.12%↓)
10 am	**50**	**998**	116 ± 1.98	**132.37 ± 2.26** (14.11%↑)	118.5 ± 2.02 (2.16%↑)	**63.89 ± 1.09** (44.92%↓)
11 am	48.4	995	111.89 ± 1.91	127.79 ± 2.18 (14.21%↑)	**122.11 ± 2.09** (9.13%↑)	**63.89 ± 1.09** (42.9%↓)
12 pm	47.7	912	88.35 ± 1.51	101.46 ± 1.73 (14.84%↑)	109.09 (9.13%↑)	55.07 ± 0.94 (37.67%↓)
01 pm	46	758	98.87 ± 1.69	98.41 ± 1.68 (0.47%↓)	118.52 ± 2.03 (23.47%↑)	60.3 ± 1.03 (39.01%↓)
Max η	–	–	–	14.21%↑	23.47%↑	37.67%↓
Max	50	998	116	132.37	122.11	63.89
Mean	47.62	906.6	102.45	112.57	116.48	57.54

The application of active cooling techniques resulted in a significant reduction in PV surface temperature. The water-spray cooling method (Panel B) demonstrated the most pronounced temperature reduction during peak irradiance periods, particularly around noon, due to the combined effects of convective heat transfer and evaporative cooling. This thermal reduction directly corresponded to the observed enhancement in output power and conversion efficiency during high-temperature intervals.

Similarly, the iron-serpentine water-cooling technique (Panel C) provided a consistent and stable temperature reduction over the operating period. Although its instantaneous cooling effect was slightly lower than that of the spray system at peak temperatures, it maintained lower average panel temperatures during morning and early afternoon hours, which explains its superior average power output observed in several measurement intervals.

In contrast, the fame-glass technique (Panel D) reduced the PV panel temperature by partially blocking direct solar radiation; however, this temperature reduction was accompanied by a substantial decrease in incident irradiance on the PV surface. Consequently, despite the lower operating temperature, the electrical output and efficiency were consistently inferior compared to both the reference and actively cooled panels.

Overall, the temperature variation analysis confirms that active cooling techniques are more effective than passive shading-based approaches in high-temperature environments. The results highlight a strong correlation between PV temperature reduction and electrical performance enhancement, validating the necessity of thermal management for PV systems operating in arid and hot climates such as Upper Egypt.

Tables 3, 4, 5 include the experimentally measured PV surface temperature values corresponding to the four analyzed cooling scenarios, which are used to interpret the observed variations in electrical power output.

**Table 4:** A reading of each panel during day work (September 15^th^, 2022).

Time	Temperature, °C	Irradiance, w/m^2^	Output power, w			
			Panel (A)	Panel (B)	Panel (C)	Panel (D)
09:00 am	45	797	109.01 ± 1.86	109.03 ± 1.86 (0.02%↑)	110.70 ± 1.89 (1.55%↑)	31.76 ± 0.54 (70.87%↓)
10:00 am	47.5	1070	100.97 ± 1.73	101.47 ± 1.73 (0.5%↑)	119.66 ± 2.04 (18.5%↑)	60.3 ± 1.03 (40.3%↓)
11:00 am	48.2	**1132**	111.89 ± 1.91	130.4 ± 2.23 (16.548%↑)	125.79 ± 2.15 (12.42%↑)	63.89 ± 1.09 (42.9%↓)
12:00 pm	**50.7**	1070	111.89 ± 1.91	**135.55** ± 2.32 **(21.15%**↑**)**	**134.16** ± 2.29 (19.9%↑)	**75.04** ± 1.28 **(32.93%**↓**)**
01:00 pm	50	1070	**117.22** ± 2	129.09 ± 2.21 (10.13%↑)	120.34 ± 2.06 (2.66%↑)	57.7 ± 0.99 (50.78%↓)
02:00 pm	48.3	965	88.35 ± 1.51	105.76 ± 1.81 (19.71%↑)	109.09 ± 1.86 **(23.47%**↑**)**	55.07 ± 0.94 (37.67%↓)
Max η	–	–	–	(21.15%↑)	(23.47%↑)	(32.93%↓)
Max	50.7	1132	117.22	135.55	134.16	75.04
Mean	48.28	1017.33	106.56	118.55	119.96	57.29

**Table 5:** A reading of each panel during day work (October 1^th^, 2022).

Time	Temperature, °C	Irradiance, w/m^2^	Output power, w			
			Panel (A)	Panel (B)	Panel (C)	Panel (D)
11:00 am	51	1118	119.7 ± 2.05	136.4 ± 2.33 (13.95%↑)	122.1 ± 2.09 (2%↑)	63.89 ± 1.09 (46.62%↓)
12:00 pm	52.5	1136	106.7 ± 1.82	130.5 ± 2.23 (22.3%↑)	111.93 ± 1.91 (4.9%↑)	72.52 ± 1.24 (32.03%↓)
04:00 pm	45	700	75.33 ± 1.2	75.27 ± 1.29 (0.08%↓)	76.24 ± 1.30 (1.2%↑)	22.49 ± 0.38 (70.14%↓)

#### Measurements of the first day

The first day is the 1^st^ of September in 2022; the reading is during the daytime from 9 am to 1 pm. Its reading is listed in Table 3 which shows the comparison between the maximum output powers of each type of panels during this day. The Panel B records the highest maximum output power, while the Panel C records the best average output power, and maximum efficiency. However, the Panel D records the worst power output.

**Fig. 10:**
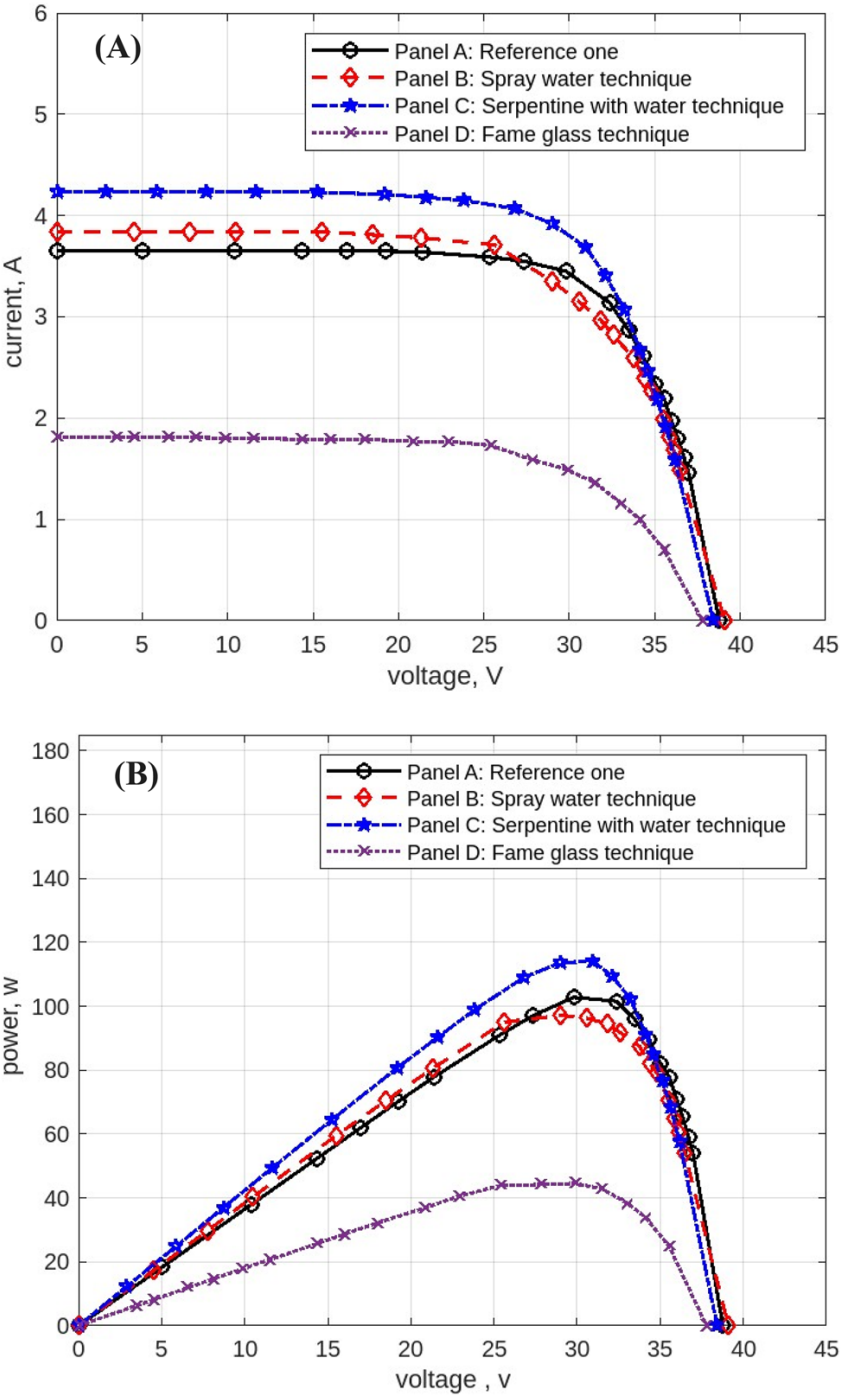
Experimental results of using different cooling techniques of same PV panels; (**A**) I-V curve, and (**B**) P–V curve; (September 1^st^, 2022; 9 am).

**Fig. 11:**
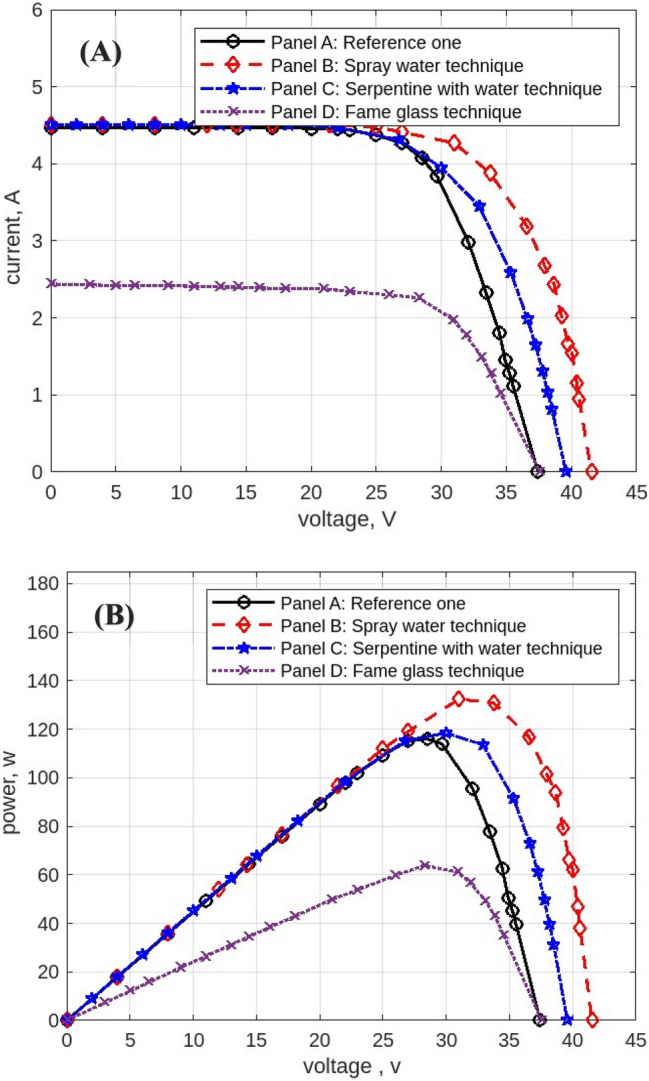
Experimental results of using different cooling techniques of same PV panels; (**A**) I-V curve, and (**B**) P–V curve; (September 1^st^, 2022; 10 am).

**Fig. 12:**
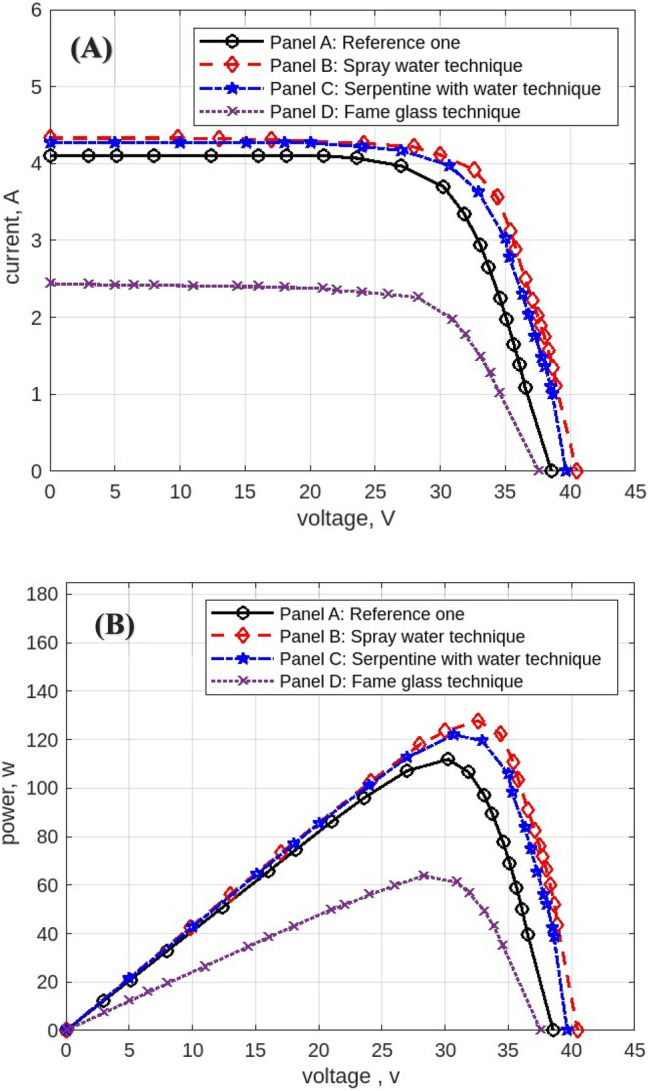
Experimental results of using different cooling techniques of same PV panels; (**A**) I-V curve, and (**B**) P–V curve; (September 1^st^, 2022; 11 am).

**Fig. 13:**
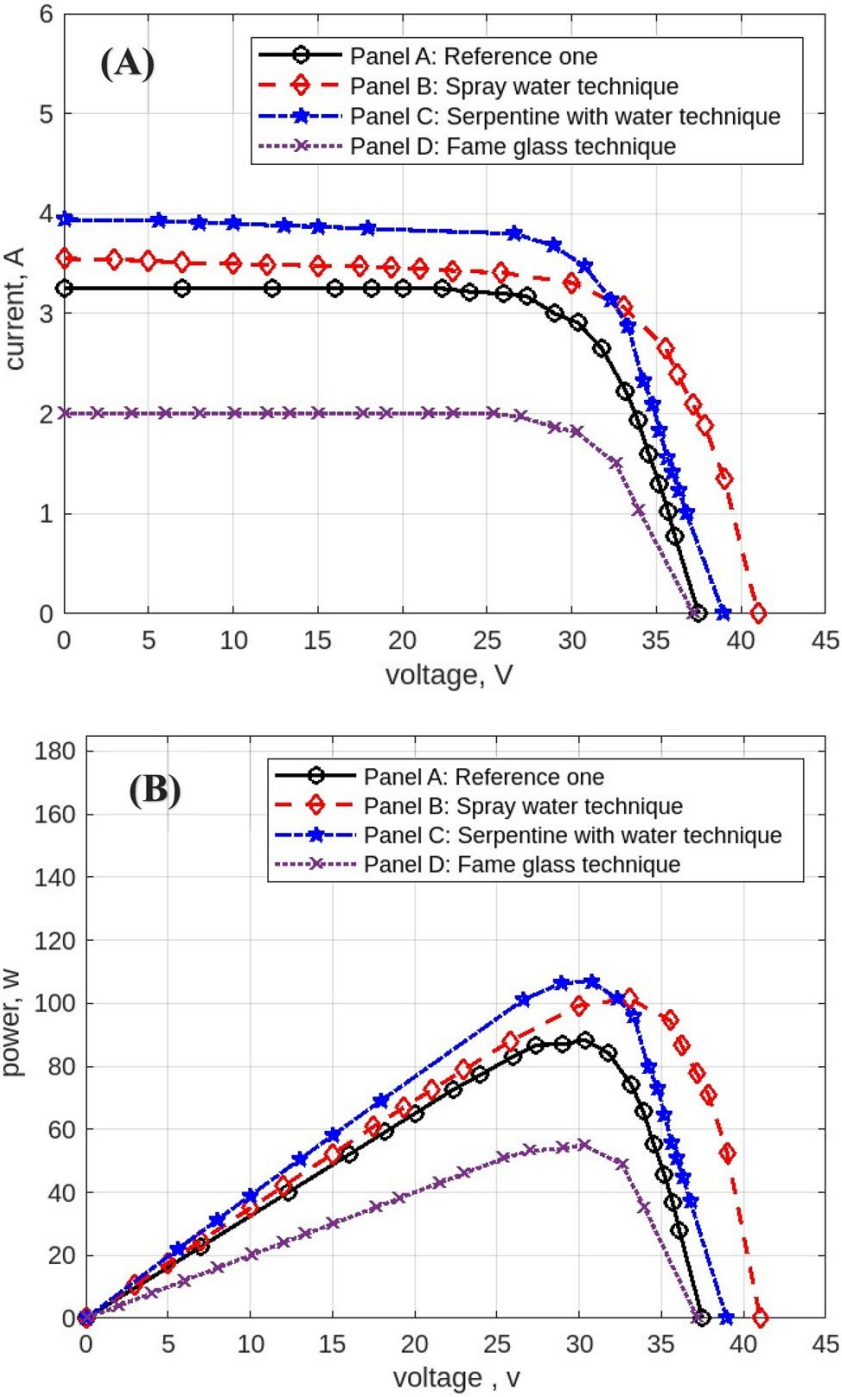
Experimental results of using different cooling techniques of same PV panels; (**A**) I-V curve, and (**B**) P–V curve; (September 1^st^, 2022; 12 pm).

**Fig. 14:**
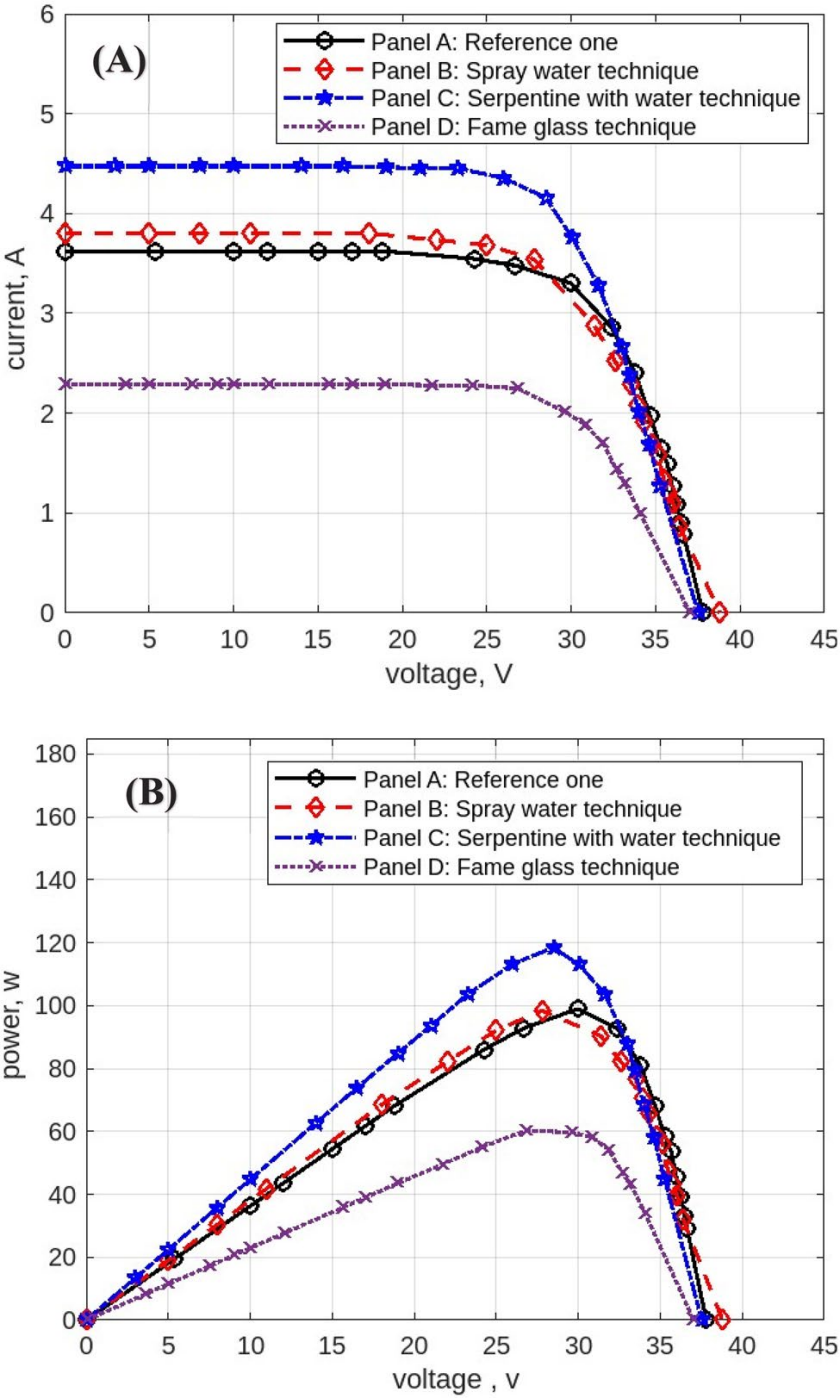
Experimental results of using different cooling techniques of same PV panels; (**A**) I-V curve, and (**B**) P–V curve; (September 1^st^, 2022; 1 pm).

The results of the experiment of the I-V and P–V curves using various cooling methods of the same PV panels at 9 am, are displayed in Fig. 10. Panel B’s efficiency improves by approximately 5%, Panel C’s efficiency increases by approximately 18%, and Panel D’s efficiency declines by approximately 54%. Then, the experimental results of the I-V and P–V of PV panels at 10 am, with a maximum irradiation of 998 w/m^2^ and maximum temperature of 50 °C, are displayed in Fig. 11. Panel B’s efficiency improves by approximately 21%, Panel C’s efficiency increases by approximately 9%, and Panel D’s efficiency decreases by approximately 41%. While Fig. 12 shows the experimental results of the I-V and P–V curves of PV panels at 11 am. Panel B’s efficiency improves by approximately 16%, Panel C’s efficiency increases by approximately 11%, and Panel D’s efficiency decreased by approximately 42%. Subsequently, at 12 pm the experimental results of PV panels’ I-V and P–V curves are displayed in Fig. 13. Panel B’s efficiency improves by approximately 16%, Panel C’s efficiency improves by approximately 25%, and Panel D’s efficiency decreased by approximately 36%. Finally, Fig. 14 shows the experimental results of the I-V and P–V curves of PV panels at 1 pm.

According to the results, on September 1^st^, the Panel B cooling panels with spray water method recorded a maximum output power of 133 w and an enhanced efficiency of 21%; on the other hand, the Panel C cooling panels with iron-serpentine with water technique recorded the best average power 117.2 w, and best efficiency of 25% at the morning. Panel B outperforms Panel C between 11 am and 12 pm., whereas Panel C outperforms Panel B at other reading times.

#### Measurements of the second day

The second day is the 15^th^ of September in 2022; the readings are during the daytime from 9 am and extend to 2 pm. In this case, the maximum temperature reading is 50 °C at 12:55 pm. Its reading is listed in Table 4 which shows a comparison between the maximum output power of each type of panel during this day. The Panel B records the highest maximum output power, while the Panel C records the best average output power, and the best efficiency. However, the Panel D records the worst power output.

**Fig. 15:**
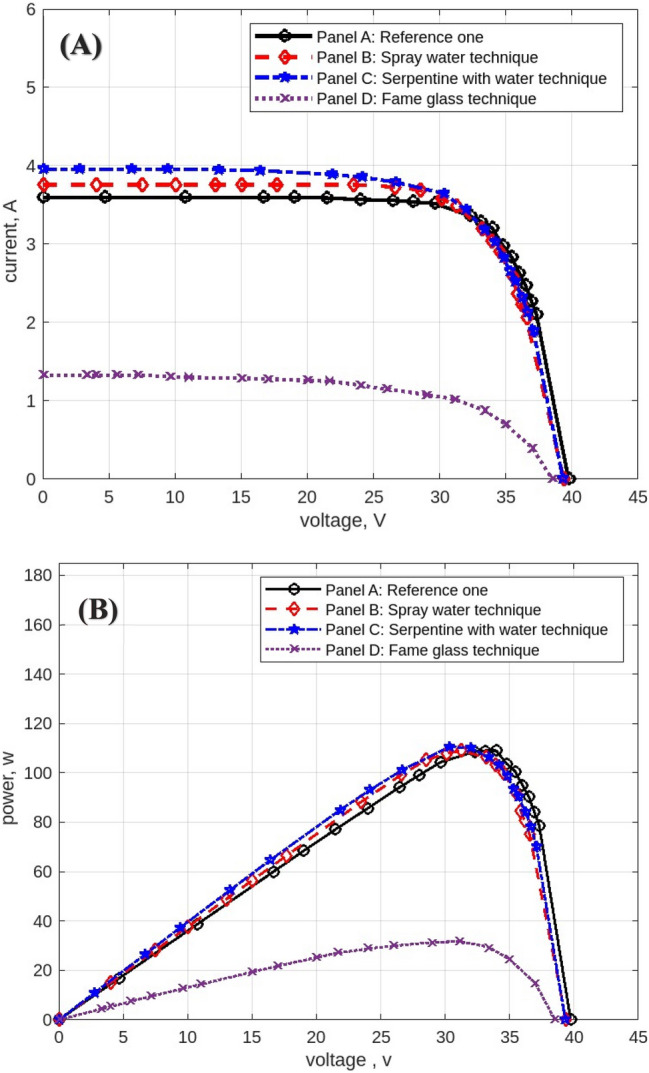
Experimental results of using different cooling techniques of same PV panels; (**A**) I-V curve, and (**B**) P–V curve; (September 15^th^, 2022; 9 am).

**Fig. 16:**
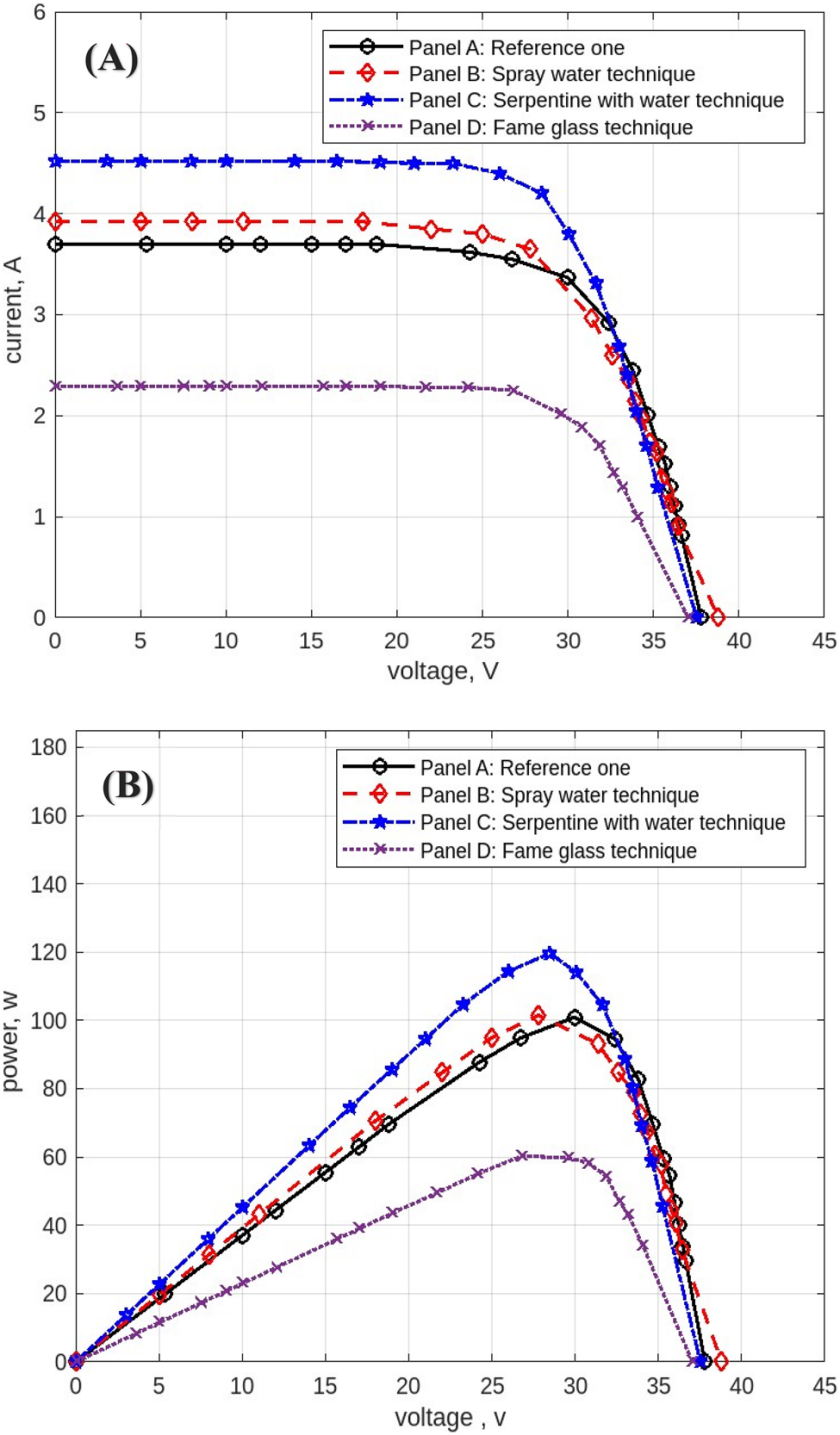
Experimental results of using different cooling techniques of same PV panels; (**A**) I-V curve, and (**B**) P–V curve; (September 15^th^, 2022; 10 am).

**Fig. 17:**
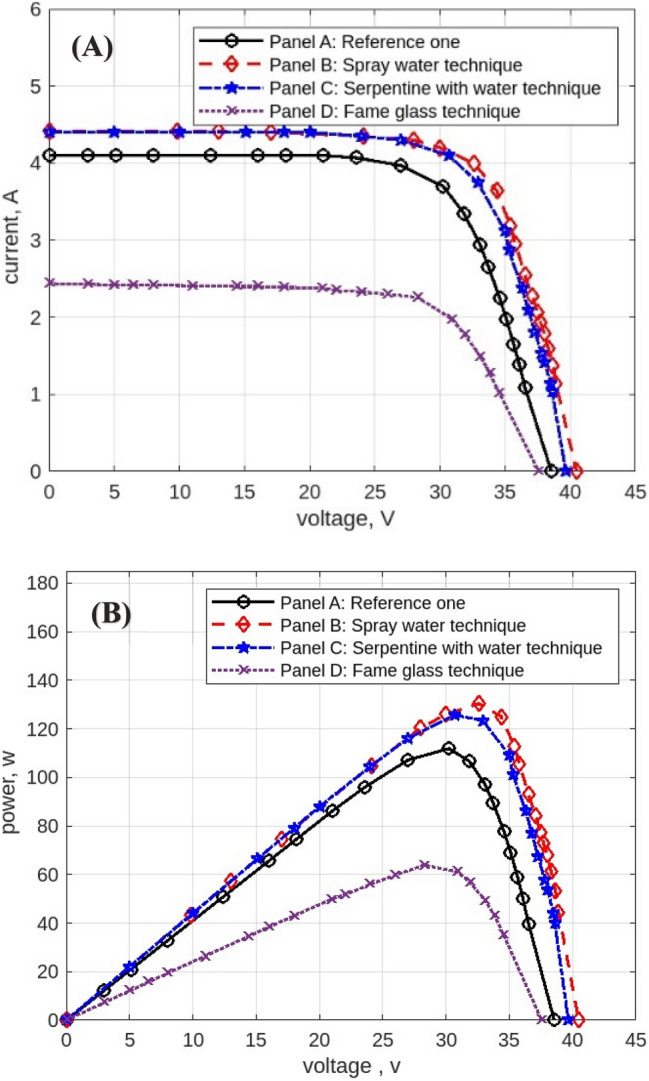
Experimental results of using different cooling techniques of same PV panels; (**A**) I-V curve, and (**B**) P–V curve; (September 15^th^, 2022; 11 am).

**Fig. 18:**
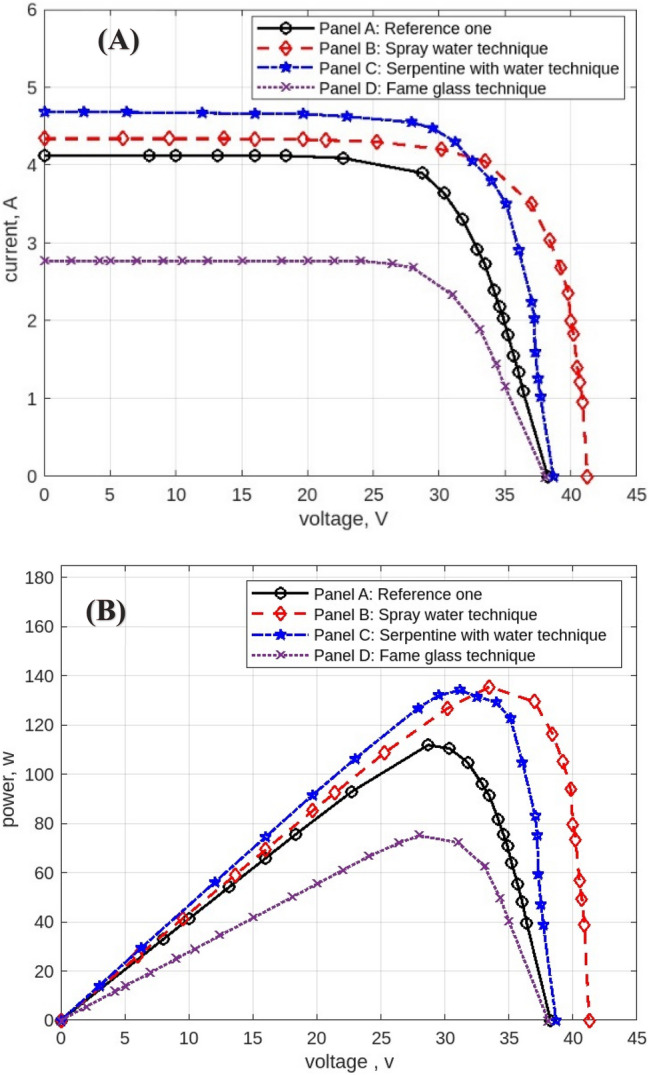
Experimental results of using different cooling techniques of same PV panels; (**A**) I-V curve, and (**B**) P–V curve; (September 15^th^, 2022; 12 pm).

**Fig. 19:**
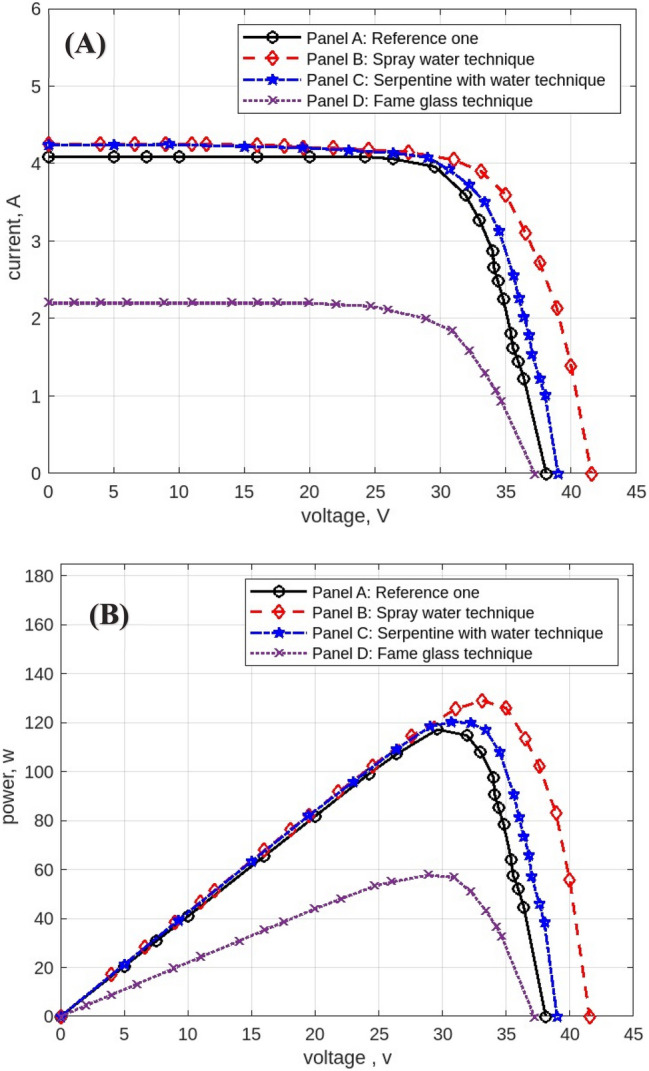
Experimental results of using different cooling techniques of same PV panels; (**A**) I-V curve, and (**B**) P–V curve; (September 15^th^, 2022; 1 pm).

**Fig. 20:**
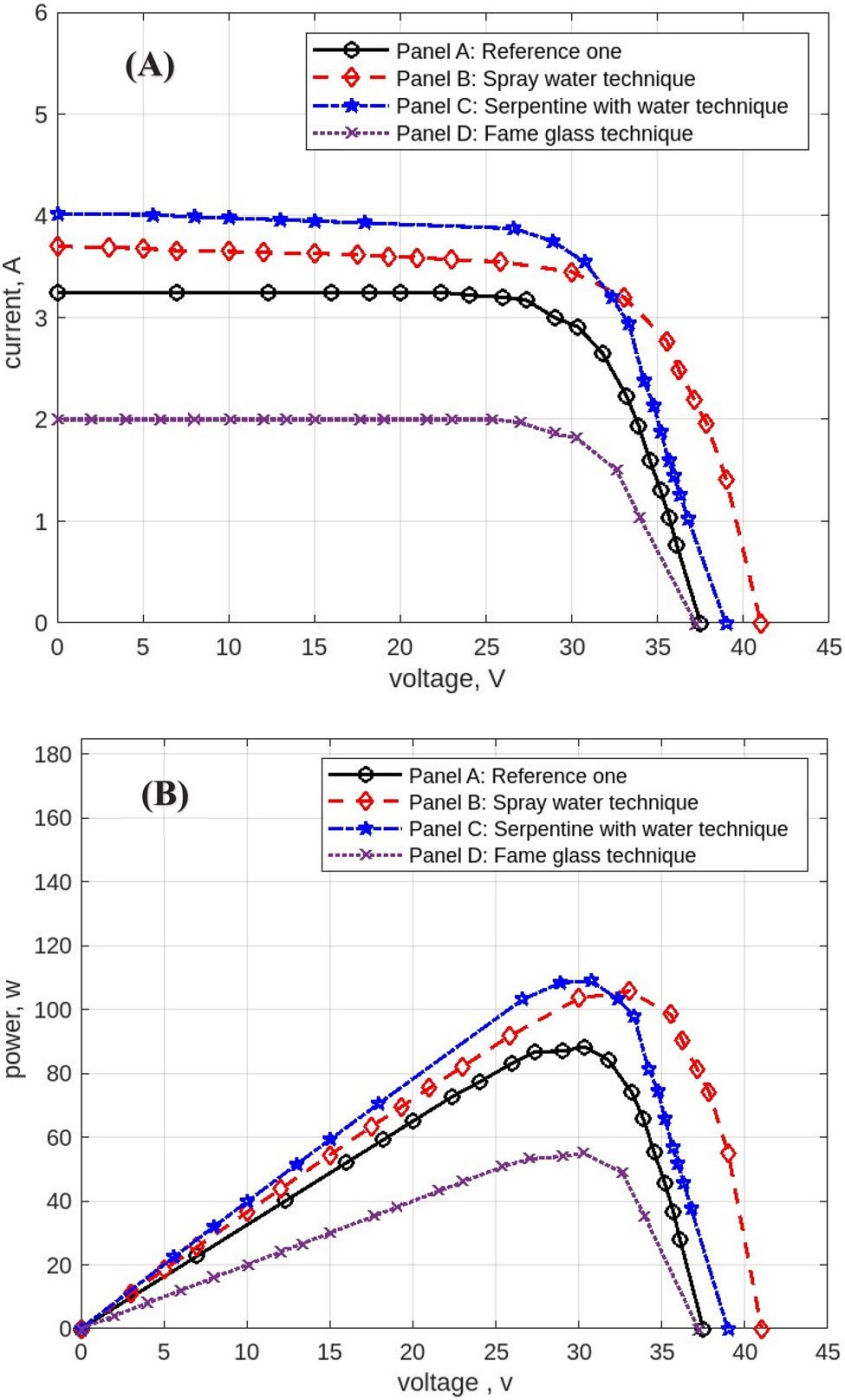
Experimental results of using different cooling techniques of same PV panels; (**A**) I-V curve, and (**B**) P–V curve; (September 15^th^, 2022; 2 pm).

The experimental results of the I-V and P–V curves utilizing various cooling methods of the same PV panels on September 15, 2022, at 9 am, are displayed in Fig. 15**.** Panel B’s efficiency gains approximately 1% over Panel A’s, Panel C’s efficiency gains approximately 2% over Panel A, and Panel D’s efficiency decreases approximately 70% less than Panel A’s reference panel. Then Fig. 16 displayed curves at 10 am. Panel B’s efficiency increases by approximately 1% compared to Panel A’s reference panel; Panel C’s efficiency increases by approximately 19% compared to Panel A; and Panel D’s efficiency decreases by approximately 40% compared to Panel A’s reference panel. Furthermore, Fig. 17 shows the experimental results of I-V and P–V curves using different cooling techniques of the same PV panels at 11 am with maximum irradiance = 1132 w/m^2^. Panel B’s efficiency increases by approximately 18%, Panel C’s efficiency increases by approximately 14%, and Panel D’s efficiency decreases by approximately 42%. While Fig. 18 shows the experimental results of I-V and P–V curves using different cooling techniques of the same PV panels at 12 pm with maximum temperature = 50.7° C. Panel B’s efficiency is increased by approximately 23%, Panel C’s efficiency is increased by approximately 21%, and Panel D’s efficiency is decreased by approximately 32%, in this reading, panels with cooling techniques records the maximum produce of power in the day. Whereas in Fig. 19 that display experimental results at 1 pm records the maximum output of daily records for reference panel A, and Panel B’s efficiency is increased by approximately 11%, Panel C’s efficiency is increased by approximately 3%, and Panel D’s efficiency is decreased by approximately 50%. Finally, Fig. 20 shows the experimental results at 2 PM. Panel B’s efficiency is increased by approximately 21%, Panel C’s efficiency is increased by approximately 24%, and Panel D’s efficiency is decreased by approximately 37%.

As shown in Fig. 19, Panel D exhibits a lower operating temperature compared to the reference single PV module. However, this temperature reduction does not translate into higher electrical power output. This behavior is attributed to the glass-frame configuration, which introduces partial shading and optical attenuation that significantly reduce the effective solar irradiance reaching the PV surface. Consequently, the irradiance loss dominates over the thermal gain, resulting in a net reduction in power output. This outcome highlights the inherent trade-off associated with passive glass-based cooling techniques in high-irradiance environments.

From these experimental results, on the 15^th^ of September, the maximum output power is produced from Panel B equal to 136 w with a maximum efficiency of Panel B at 23%. This recording in the maximum temperature record in reading, while Panel C records the best average power in the day equal 120.5 w, and the best efficiency 24% at after noon. Panel C did not record better efficiency in the mid-day time. On the other hand, Panel D records the worst solution to produce power.

**Fig. 21:**
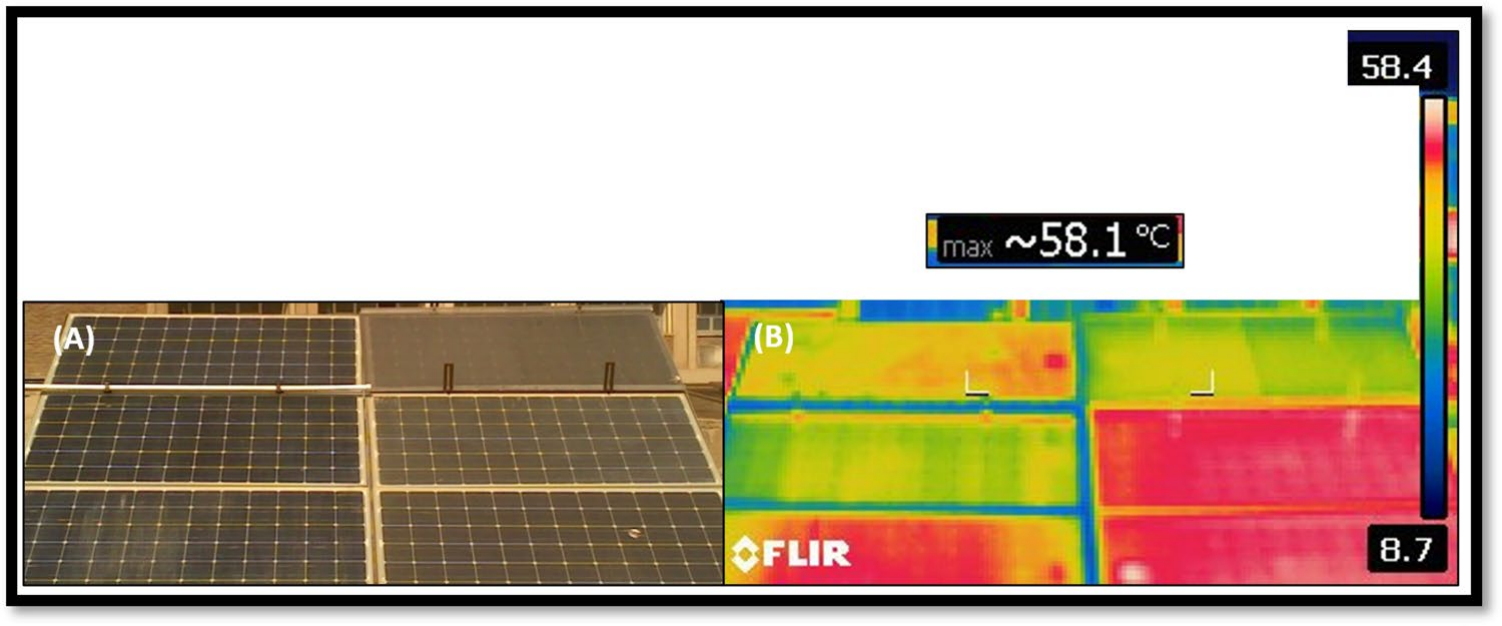
Thermal effect resulting of different cooling techniques of PV Panels; (**A**) normal picture and (**B**) thermal picture. (September 15^th^, 2022; 12:55 pm).

The thermal impact of various PV panel cooling systems is shown in Fig. 21 at 12:55 PM with an irradiation of 1070 w/m^2^ and an ambient temperature of 50 – 60 °C. The reference panel (Panel A) has high temperatures that reach 55–65 °C. Various cooling procedures are used to lower the temperatures, including water spraying (Panel B), iron-serpentine (Panel C), and fame-glass (Panel D). Under the high-temperature case, the effective solutions to reduce the temperature of PV panels are water spraying and fame-glass. However, fame-glass technique shaded the sunlight on the PV, which led to reduced production power.

#### Measurements of the third day

The third day is the 1^st^ of October in 2022, and the reading during the daytime is extended to 4 pm. To study the effect of different cooling systems before sunset, which shows the comparison between the maximum output power of each type of panel during this day. The Panel B records the highest maximum output power, while the Panel C records the best average output power. However, the panel D records the worst power output.

**Fig. 22:**
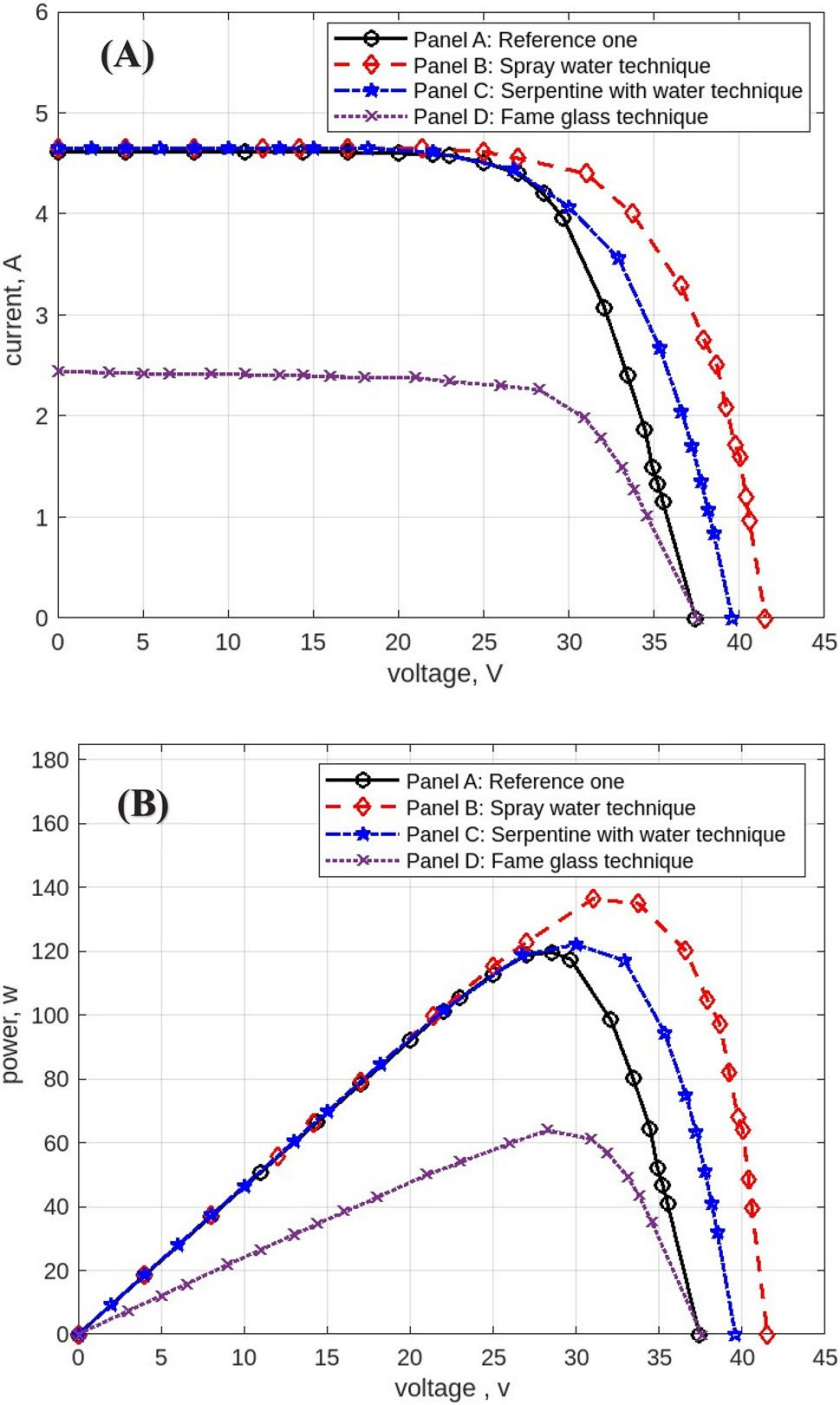
Experimental results of using different cooling techniques of same PV Panels; (**A**) I-V curve, and (**B**) P–V curve; (October 1^st^, 2022; 11 am).

**Fig. 23:**
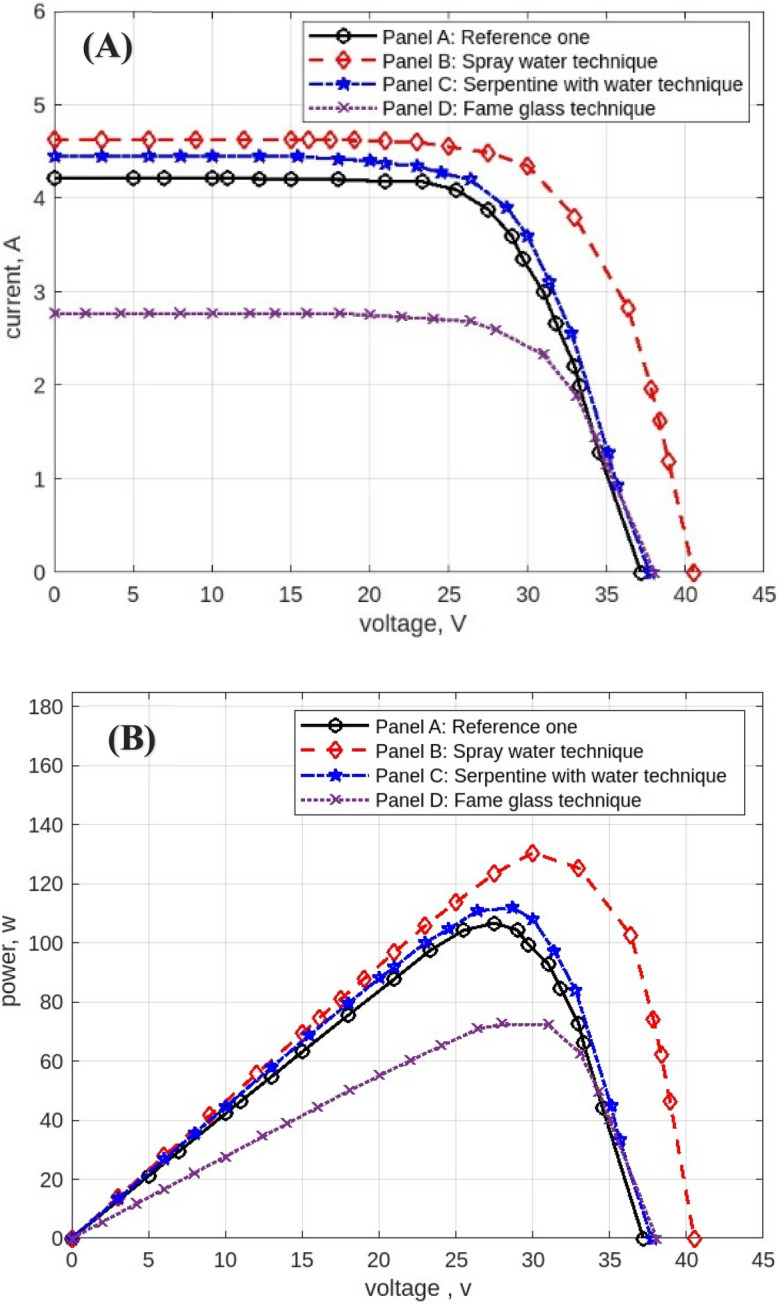
Experimental results of using different cooling techniques of same PV Panels; (**A**) I-V curve, and (**B**) P–V curve; (October 1^st^, 2022; 12 pm).

**Fig. 24:**
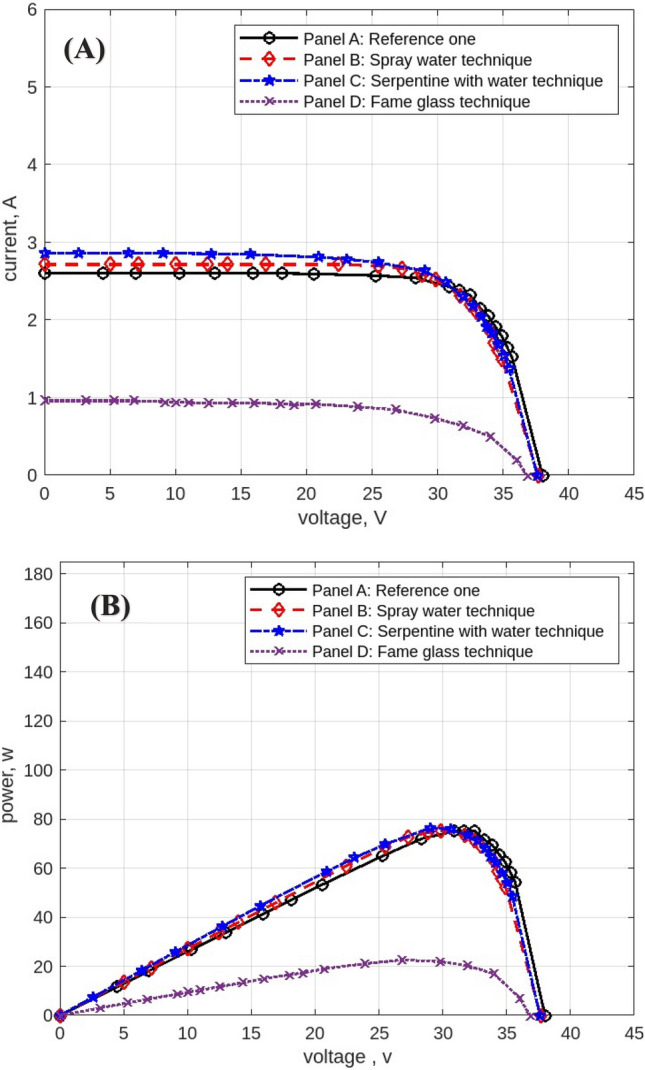
Experimental results of using different cooling techniques of same PV Panels; (**A**) I-V curve, and (**B**) P–V curve; (October 1^st^, 2022; 4 pm).

Utilizing various cooling strategies on the same PV panels. Figure 22 displays the experimental findings of the I-V and P–V curves at 11 am, In comparison to Panel A, the efficiency of Panel B using the spray-water technique increases by approximately 15%. In Panel C, the efficiency of the panel increases by approximately 3% over the reference panel (Panel A). In Panel D, the efficiency of the panel decreases by approximately 46% less than in Panel A when using the serpentine with water technique. Figure 23 displays the experimental findings of the I-V and P–V curves at 12 pm. Panel B’s efficiency increases by approximately 24% compared to Panel A’s reference panel; Panel C’s efficiency increases by approximately 6% compared to Panel A; and Panel D’s efficiency decreases by approximately 31% compared to Panel A’s reference panel. At 4 pm, Fig. 24 displays the experimental findings of the I-V and P–V curves. Compared to the reference panel (Panel A), the efficiency of panel B rises by around 0.1%; on the other hand, the efficiency of Panel C rises by approximately 2%; and Panel D, with an approximate decrease in efficiency of 68%.

As proven by the results, it is preferable to use the Serpentine with water approach (Panel C) rather than alternative methods for cooling solar PV panels at low temperatures. Using the spray water method (Panel B) is a better way to cool during high temperatures than the serpentine with water approach (Panel C). The Fame glass approach (Panel D) not recommended to use since its low power production in all measurements.

It should be noted that the observed performance enhancement under the different cooling configurations is mainly reflected in increased electrical power output due to reduced operating temperature. While power gains are significant, the corresponding conversion efficiency improvements are reported separately and remain comparatively modest, as summarized by the maximum efficiency values presented in Tables 3 and 4.

#### Impact of fame-glass and clamp-induced shading on PV performance

Although the fame-glass configuration (Panel D) resulted in a noticeable reduction in PV surface temperature, the experimental results presented in Tables 3, 4, 5 reveal a substantial decrease in output power that exceeds what can be explained by temperature effects alone. This behavior indicates the presence of additional loss mechanisms beyond thermal influence.

One major contributing factor is partial shading caused by the mechanical clamps used to secure the glass frame, as clearly visible in the thermal image shown in Fig. 6. These clamps obstruct a portion of the incident solar radiation, leading to localized shading and non-uniform irradiance distribution across the PV surface. Such shading effects are known to significantly reduce PV output power, even when the shaded area is relatively small.

Furthermore, the fame-glass layer introduces inherent optical losses due to reflection, refraction, and attenuation of incoming solar radiation, which reduce the effective irradiance reaching the PV cells. Consequently, despite operating at a lower surface temperature, Panel D consistently exhibited the lowest electrical performance among all tested configurations.

These combined effects explain the disproportionately large power reduction observed in the fame-glass scenario and confirm that this technique does not provide a fair thermal–electrical trade-off when compared to active cooling methods. Therefore, the fame-glass approach is not recommended for practical PV cooling applications, especially in high-irradiance and hot climatic conditions.

The noticeably lower power output recorded for Panel D is primarily attributed to optical losses caused by partial shading from the glass-frame mounting clamps rather than thermal effects. Although the glass-frame configuration achieved a measurable reduction in PV surface temperature, the associated irradiance attenuation and localized shading significantly limited the electrical output. Consequently, Panel D is considered as a reference passive shading case, and its results are discussed qualitatively to highlight the limitations of glass-based cooling approaches under practical outdoor installation conditions, rather than as a directly comparable alternative to the active cooling techniques.

The hourly variation of PV surface temperature for the investigated cooling configurations is presented to illustrate the dynamic thermal response of the modules under outdoor conditions. These temperature trends directly influence the corresponding electrical power output and efficiency behavior throughout the day. While efficiency variations are not plotted separately for each hour, their impact is inherently captured through the measured power output and summarized by the maximum efficiency values reported for each configuration.

The fame-glass technique demonstrates that temperature reduction alone is insufficient to guarantee performance enhancement when accompanied by significant irradiance attenuation.

The findings of this study provide preliminary insights into the applicability of PV cooling techniques, while long-term field validation remains a necessary step for large-scale deployment.

### Cost analysis

In this section, the cost analysis is presented for proposed cooling techniques. This analysis determined components with their capital and operation costs.

The capital cost is listed in .

Table 6, the item prices are from amazon expected the serpentine and fame glass sheet from local market. This table shows the spray water cooling system is the most effective system, and fame glass is the simplest. Serpentine has the high capital cost in its system.

**Table 6:** The capital cost of each cooling system.

Spray water		serpentine		Fame glass	
Item	cost $	item	cost $	item	cost $
Pump 45w	9	Pump 45w	9	Glass	34
Tank 50L	5.8	Tank 50L	5.8	Clamp *4	1.2
PVC tubes 1.5 m	1	Serpentine	50	–	–
Hose	3	hose	6	–	–
Total	18.8	Total	50.8	Total	35.2

Table 7 has tabulated the operation cost for various operation cost, fame glass as a passive system hasn’t needed to energy or water. While the cost in the active system, Spray water is effective in reduced energy consumption. However, it needs to water continuously. In the other side the serpentine cooling system most effective in saving water since it’s a closed cycle. Serpentine uses more energy because pump always works.

**Table 7:** The operation cost of each cooling system.

Item	Spray Water	Serpentine	Fame glass
Pump operation	45*(5*4/60) = 15 W/hr	45W/hr	0
Water	2*(5*4) = 40 L / hour and per day 260 Liter	50 for whole operation closed system	0

The main key to choose the suitable cooling system is the environment, is the water is available and with low cost, or not, then the ambient temperature degrees.

## Limitations of the study

Despite the promising results obtained in this study, several limitations should be acknowledged. First, the experiments were conducted on a single PV module under specific outdoor environmental conditions, which may limit the generalization of the results to larger PV systems. Second, the study focused on a limited number of cooling techniques and a specific experimental configuration. Additionally, the experiments were carried out during a defined testing period, and seasonal variations were not fully considered. Future work may extend this study by investigating long-term performance under different climatic conditions and applying the proposed cooling techniques to larger PV installations.

## Conclusion

Photovoltaic (PV) panels in normal operation without cooling as Panel (A), the highest efficiency of the panel is at noon, it is in the middle at 9 and 10 am and the lowest efficiency is at two in the afternoon. Since the effect of high ambient temperature. The fame glass technique has reduced the temperature of PV, whereas it reduced the efficiency all the time as shown in results in Panel (D), since this technique reduced the irradiance also.

The water spray technique has the highest efficiency of the panel at noon. But the efficiency is often low in the early morning and late afternoon. The iron serpentine technique has the highest efficiency most of the time except in the early morning and late afternoon as proven by results in Panels (B) and (C).

The results demonstrate that active cooling techniques, particularly spray and serpentine cooling, lead to noticeable improvements in PV electrical performance due to effective reduction of operating temperature. In contrast, the glass-frame technique, although successful in lowering the module temperature, results in a reduction in electrical output because of decreased incident irradiance. These findings confirm that temperature reduction alone does not guarantee efficiency enhancement, and that the balance between thermal cooling and irradiance availability is critical for achieving net performance gains.

This study has been recommended using the Serpentine with water technique, at low temperature. Whereas the water spray cooling technique is recommended at the high temperature. Finally, this study hasn’t been recommended the Fame-glass technique.

## Future work

- Other cooling techniques can be studied in future works such as Active air-cooling, PCM, Continuous water spraying cooling, and Back-water spraying cooling.
- Testing the spray system with various flowrate and operation time to determine other settings.
- Testing system of serpentine with different (on / off) times, and cooling the water with other method than icing to provide the more effective method and economic cost.
- Using advanced devices to measure VI and PV curves.
- Using individual temperature sensors to measure each panel temperature.
- Using wind sensor to measure the effect of wind speed.

## Data Availability

The datasets used and/or analyzed during the current study available from the corresponding author on reasonable request.
